# Elucidation of the biosynthesis pathway and heterologous construction of a sustainable route for producing umbelliferone

**DOI:** 10.1186/s13036-019-0174-3

**Published:** 2019-05-22

**Authors:** Yucheng Zhao, Xiangyun Jian, Jialin Wu, Wanchun Huang, Chuanlong Huang, Jun Luo, Lingyi Kong

**Affiliations:** Jiangsu Key Laboratory of Bioactive Natural Product Research and State Key Laboratory of Natural Medicines, School of Traditional Chinese Pharmacy, China Pharmaceutical University, Nanjing, 210009 People’s Republic of China

**Keywords:** Coumarins, Biosynthetic pathway, Metabolic engineering, Protein engineering, Synthetic biology

## Abstract

**Background:**

Coumarins play roles in many biological processes. *Angelica decursiva* is one of the major sources of coumarins in China. Due to increasing demand for coumarins in the marketplace, traditional extraction from plants is now considered economically insufficient and unsustainable. Microbial synthesis is a promising strategy for scalable production of coumarins. However, the biosynthetic pathway of coumarin remains poorly understood, and even more, the genes associated with this process have not been characterized in *A. decursiva*.

**Results:**

RNA-seq was employed to elucidate the umbelliferone biosynthetic pathway. The results indicated that three enzymes, phenylalanine ammonia-lyase (PAL), 4-Coumarate: Coenzyme A Ligase (4CL), and *p*-coumaroyl CoA 2'-hydroxylase (C2’H) were involved in umbelliferone biosynthesis. Using the cloned genes, we generated a synthetic biology based microbial cell factory that produces coumarins from tyrosine utilizing *Rhodotorula glutinis* tyrosine ammonia lyase (RgTAL) to bypass cinnamic acid 4-hydroxylase (C4H). With metabolic engineering strategies, we deleted *prephenate dehydratase (pheA), anthranilate synthase (trpE)* and *transcriptional regulatory protein (tyrR)* and overexpressed six related genes involved in tyrosine biosynthesis, to drive the carbon flux from tyrosine. To overcome the limitation of 4CL, a virtual screening and site-specific mutagenesis-based protein engineering approach was applied. In addition, induction/culture conditions and different ions were employed to further improve the yield of umbelliferone. Finally, a yield of 356.59 mg/L umbelliferone was obtained.

**Conclusions:**

The current study elucidated the umbelliferone biosynthesis pathway in *A. decursiva*. The results also demonstrated the feasibility of integrating gene mining with synthetic biology techniques to produce natural compounds.

**Electronic supplementary material:**

The online version of this article (10.1186/s13036-019-0174-3) contains supplementary material, which is available to authorized users.

## Background

Coumarin, which has a 2H-1-benzopyran-2-one core structure, is widely distributed in the Umbelliferae, Fabaceae, Rosaceae, Rutaceae, and Saxifragaceae [1]. Apart from their role in environmental adaptation, coumarin derivatives have also been demonstrated to have anti-inflammatory, anticancer, antioxidant, and anti-hyperglycaemic activities [2–6]. Hence, securing the supplies of these compounds from medicinal plants has been a long-standing practice. *A. decursiva* is one of the main sources of coumarins in China and is listed as a special coumarin resource in the current Pharmacopeia of China [7]. However, low abundance and season- or region-dependent sourcing limit its widespread application. Producing coumarin via solvent extraction or soil excavation is considered harmful to the environment [8, 9]. In addition, the complexity and multiple chiral centers of these compounds limit their production via industrial de novo chemical synthesis [10]. Owing to these issues, pharmaceutical companies and scientists are seeking alternative methods. An economical and environmentally friendly production platform and/or approach appear to be urgently needed.

The use of engineered microorganisms in metabolic engineering is rapidly emerging as a promising biotechnological technique for resolving these issues. With this technology, it is possible to design and reconstruct a synthetic route in microorganisms for producing natural products [11–13]. A comprehensive understanding of structural and biochemical properties of enzymes involved in natural product biosynthesis is a prerequisite for using such methods. However, the coumarin biosynthetic pathway has not yet been completely resolved. Even more, genes associated with coumarin biosynthesis have not been cloned in *A. decursiva*. The rapidity and efficiency of gene discovery has improved dramatically, due to advances in next-generation sequencing technology (NGS), such as Roche/454 and Illumina HiSeq platforms, and annotation information available in public databases such as the National Center for Biotechnology Information (NCBI) [14, 15]. Hence, we could use RNA-seq data to identify the genes involved in coumarins biosynthesis and construct a microbial cell factory to produce umbelliferone for it formed the core structure of other kinds of coumarins [3]. However, issues such as low yield and the high cost of precursor chemicals associated with this approach make it commercially unfeasible [16, 17]. Hence, systematic construction of a sustainable route for large scale production compound using inexpensive and readily available materials is a challenge that needs to be addressed [18].

Among strategies used for successful development of industrial microbial strains, enzyme selection, pathway optimization/reconstruction, and cofactor/precursor availability are considered to be most important [18]. In enzyme selection, choosing candidate enzymes which facilitate the completion of a target route spans the traditional scope of consideration. However, refining the catalytic efficiency of target enzymes via enzyme and protein engineering is also needed [19]. In pathway optimization/reconstruction, the overall project design, host strain selection, rerouting and optimization of metabolic fluxes, optimization of microbial culture conditions and scale-up fermentation need to be considered. However, most work merely focuses on the design of a metabolic pathway to produce target compounds (for example, alkaloid-based drugs), and little attention is paid to systematic robustness of the pathway or its synthetic ability to improve the yield of compounds [20]. In addition, the two-step culture process and the tyrosine/precursor supplement may substantially limit large-scale production of metabolites via fermentation [21]. Hence, systematic improvement of precursor supplies and pathway robustness may further enhance the yield of target compounds (for example, flavonoids) [21].

In this study, RNA-seq dataset and functional verification were initially employed to elucidate the umbelliferone biosynthetic pathway. Next, the genes involved in umbelliferone biosynthesis were constructed in prokaryotic expression vectors and introduced into *Escherichia coli* to produce the umbelliferone. In this process, gene knockout and overexpression were conducted to construct a L-tyrosine platform to ensure precursor supplementation. In order to bypass the 4CL limitation, virtual screening and site-specific mutagenesis-based protein engineering were applied. In addition, systematic optimization, specifically of medium, induction and culture conditions and the addition of different ions were employed to further improve the yield of umbelliferone. Finally, a total umbelliferone concentration of 356.59 mg/L was obtained. In summary, we elucidated the coumarin biosynthesis pathway and conducted heterologous construction of a sustainable route for umbelliferone production. The study also indicated that a combination of metabolic pathway optimization and protein engineering may be effective in producing umbelliferone.

## Results

### Candidate gene mining using transcriptome sequencing and similarity search

**Fig. 1:**
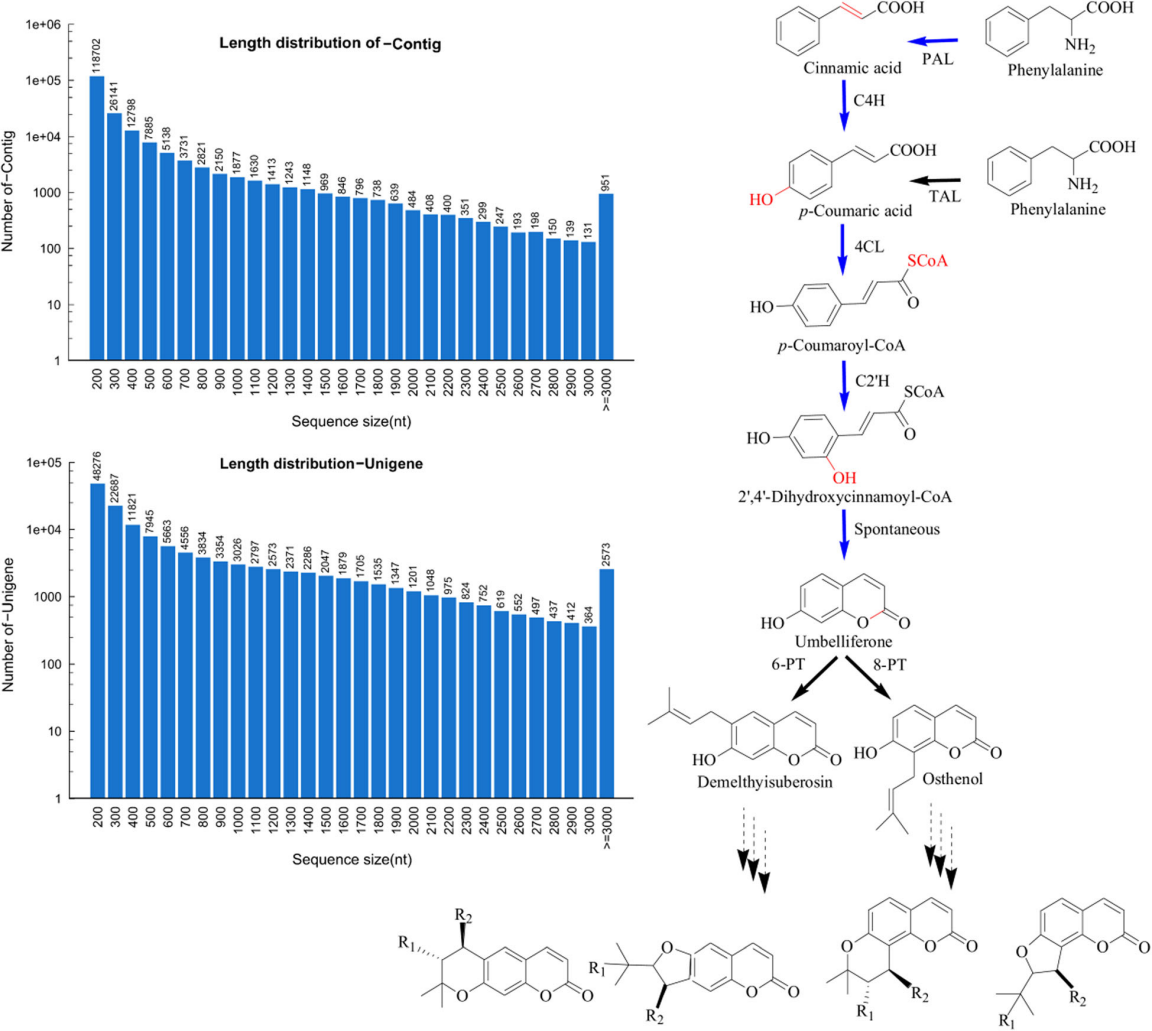
De novo assembly of the transcriptome dataset of *A. decursiva* (left) and its putative coumarin biosynthetic pathway (right). PAL, phenylalanine ammonia lyase; C4H, cinnamate 4-hydroxylase; TAL, tyrosine ammonia lyase; 4CL, 4-coumarate: coenzyme A ligase; C2’H, *p*-coumaroyl CoA 2'-hydroxylase; PT, prenyltransferase

To mine candidate genes involved in the umbelliferone biosynthesis, the transcriptome dataset of the *A. decursiva* was constructed for the first time (NCBI accession number PRJNA360870), due to the absence of information on genes related to *A. decursiva*. This produced approximately 49 million clean reads and 139,956 unigenes (194,616 contigs) with a mean length of 639 nt and a N50 of 1309 nt following de novo assembly (Fig. 1). Considering that pyranocoumarins, which originating from umbelliferone, are the main chemical constituents of *A. decursiva*, its biosynthetic mechanism was estimated according to previous reports (Fig. 1) [3, 22, 23]. Although there are no reports on the complete biosynthesis mechanism of umbelliferone, the proposed pathway, which had been functionally appraised at each step previously in other plants, seems irrefutable [24–26]. As shown in Fig. 1, the umbelliferone biosynthesis pathway was initiated by PAL, following which C4H was employed to yield *p*-coumaric acid [24]. Afterwards, 4CL was needed to produce *p*-coumaric CoA, that may be used as an intermediate to produce various compounds.

(lignin, flavones, and coumarins) [26]. Of these compounds, coumarins are produced by the formation of umbelliferone via C2’H. Next, a local BLAST search was performed to identify candidate genes involved in umbelliferone biosynthesis from our transcriptome dataset. The templates used for local BLAST search (Table 1) were from Umbelliferae plant *Peucedanum praeruptorum* (PpPAL, Pp4CL, PpC2’H), *Angelica sinensis* (AsPAL), *Petroselinum crispum* (PcC4H, Pc4CL1), *Angelica gigas* (AgC4H) and *Pastinaca sativa* (PsC2’H). And they were all functionally confirmed [25–27]. According to the identities and E-Values of the BLAST results, 13.

**Table 1:** The candidate genes involved in umbelliferone biosynthesis

Gene name	Template name (accession number)	Gene ID	Size	Score	E- Value	Identities	CDS
*PAL*	PpPAL (AYJ76815)/AsPAL (AJW77399)	*CL5684.Contig2*	2445	1347	0.0	95	7–719
		*CL5684.Contig1*	1510	950	0.0	95	159–661
		*CL5684.Contig3*	491	307	2e-83	95	138–303
		*Unigene17753*	555	281	2e-75	73	540–719
*C4H*	PcC4H(Q43033)/ AgC4H(AEA72281)	*Unigene23067*	973	539	e-153	100%	1–269
		*Unigene23068*	849	486	e-137	100%	269–505
		*Unigene24470*	1667	265	8e-71	33%	18–486
*4CL*	Pp4CL(APU52013)/ Pc4CL1(P14913)	*Unigene35034*	2074	1053	0.0	97%	1–543
		*Unigene34377*	1892	934	0.0	84%	8–543
		*Unigene33668*	1969	339	5e-93	37%	12–532
*C2’H*	PpC2’H (ASR80916)/ PsC2’H (APP94171)	*CL2481.Contig2*	1315	701	0.0	98	1–350
		*CL2481.Contig1*	800	368	e-102	75	1–236
		*Unigene29914*	1083	296	2e-80	44	1–330

unigenes/transcripts were selected as candidates, 7 of which may be true candidates (Table 1). For example, *Unigene23067* (or *Unigene23068)* has 100% identity with *PcC4H* in amino acids 1–269 (or 269–505 in *Unigene23067*), indicating that it may encode a candidate C4H in *A. decursiva.* However, *Unigene24470* is not the target gene for it has a low identity to the template (33%, Table 1). *CL2481.Contig2/Unigene35034* were completely aligned to *PpC2’H/Pp4CL*, and *Unigene23067/23068* could be linked to a single gene for which we obtained the full-length coding sequence (CDS) (Table 1).

### Functional characterization of the candidate genes

**Fig. 2:**
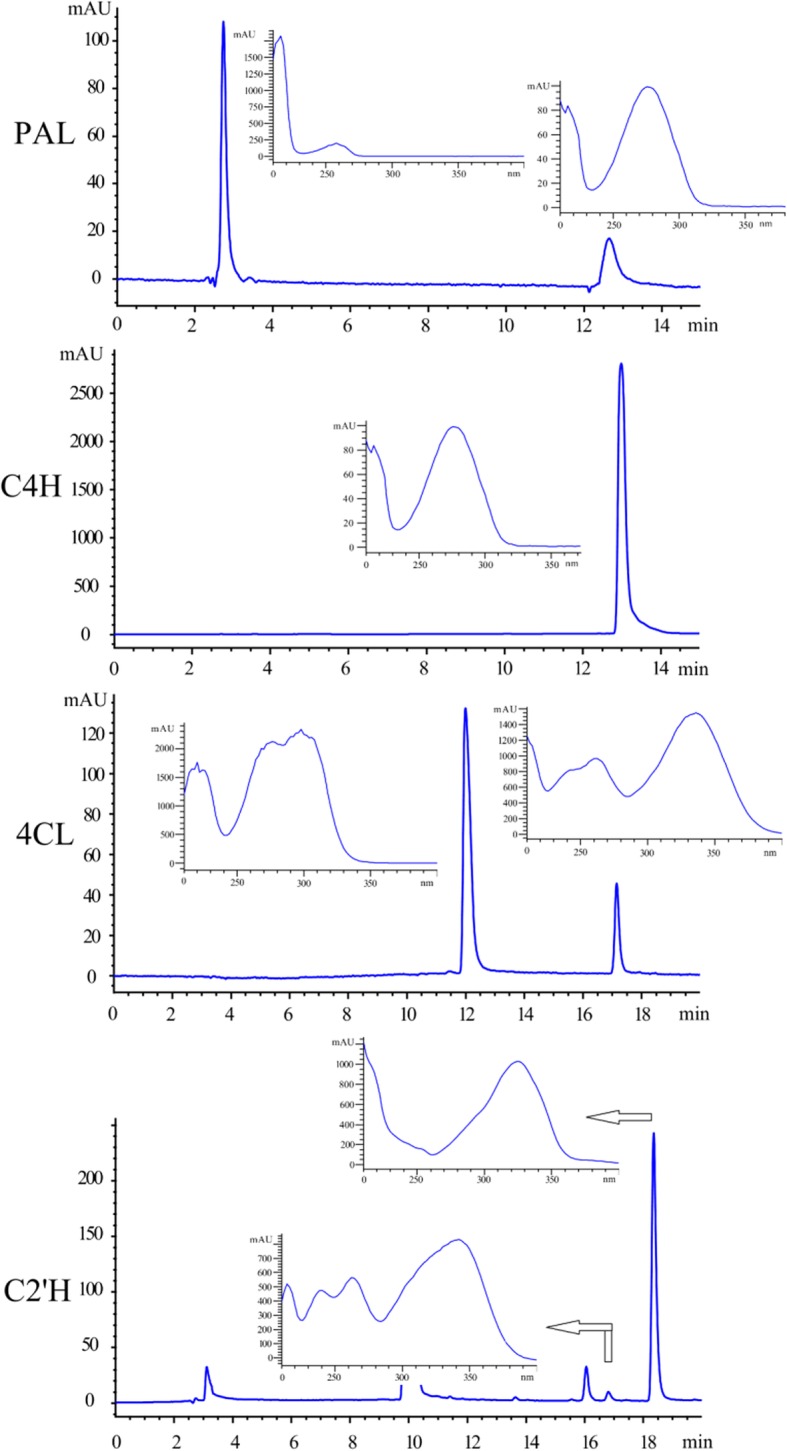
Enzymatic characteristics of AdPAL, AdC4H, Ad4CL and AdC2’H expressed in *E. coli.* Ultraviolet absorption spectra of substrates and their products are also shown beside the corresponding peak

To determine their function, deduced full-length CDSs of *AdC4H*, *Ad4CL*, *AdC2’H* and the rapid amplification of cDNA ends (RACE) product of *AdPAL* were amplified from *A. decursiva* cDNA. Following sequencing, the genes were introduced into pET28a under the T7 promoter and 6*His tag. Then, the proteins were expressed and purified for enzymatic activity on their target substrates. As a result, all enzymes except C4H readily yielded additional peaks corresponding to their expected products (Fig. 2). The data indicated that the selected genes were indeed the target genes involved in umbelliferone biosynthesis. Although its activity was not resolved, we propose that *Unigene23067/23068* is the true *C4H* because there is only one *C4H* transcript in the transcriptome dataset. In addition, nearly 100% identity with *PcC4H* also indicated that it encode *AdC4H*. Functional expression of CYP450s in prokaryotes, such as *E. coli*, is difficult due to the absence of compartmentalized organelles and membrane structure, and the fact that electrons cannot be supplied from reduced coenzyme II [28]. Hence, additional peak was not observed, and other expression systems, such as yeast, may be needed to further *C4H* expression. Finally, the 4 genes were submitted to NCBI with the accession numbers of MK350248, MK350249, MK350250 and MK350251 for *AdPAL, AdC4H, Ad4CL* and *AdC2’H*, respectively.

### Heterologous construction of a synthetic biology route for umbelliferone production

**Fig. 3:**
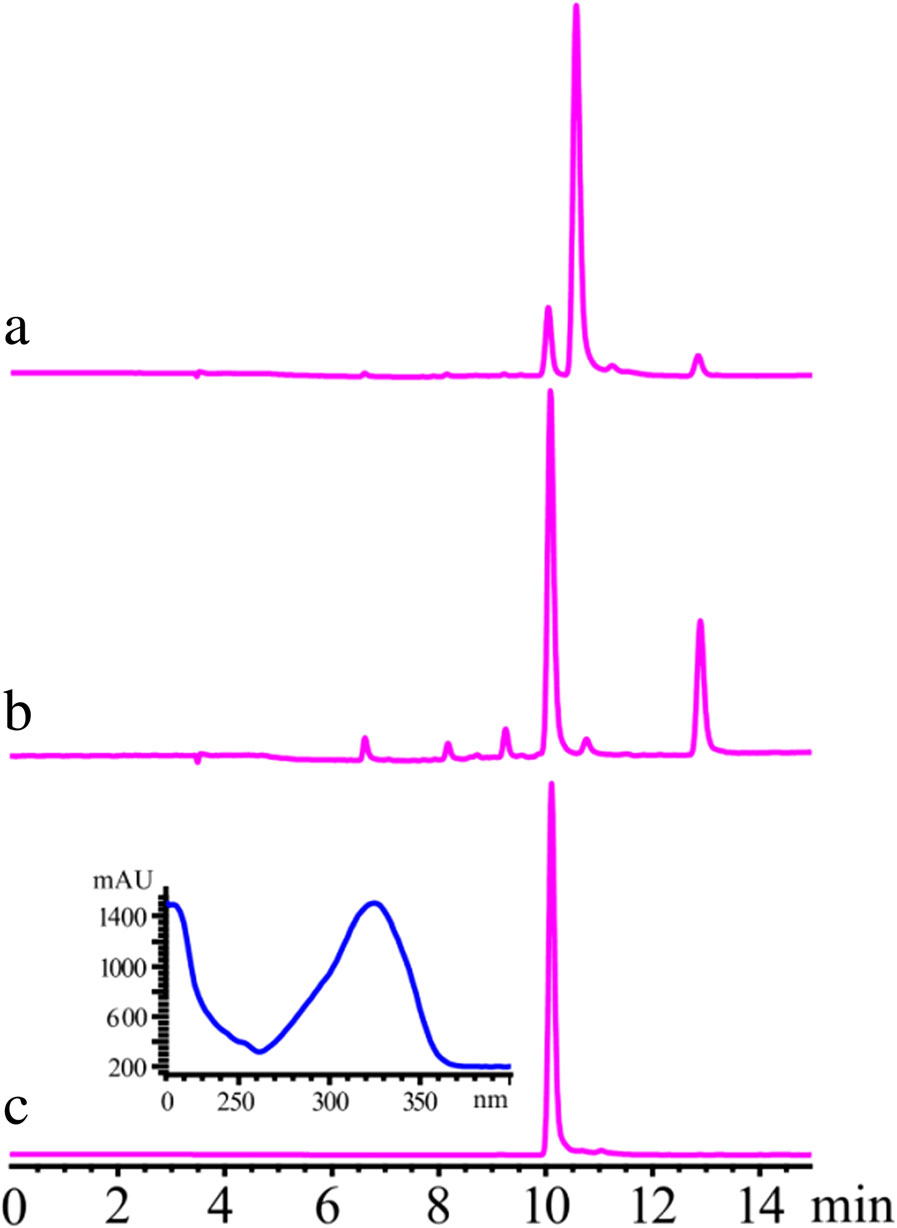
Heterologous construction of a synthetic biology route for umbelliferone production. (**a**), The first attempt at heterologous construction of a synthetic biology route for umbelliferone production; (**b**), umbelliferone production with the optimized conditions; (**c**), umbelliferone standard

Umbelliferone, which is a processed product of coumarin biosynthesis, is difficult to trace because it may be converted by prenyltransferase or other downstream enzymes to produce umbelliferone derivatives (Fig. 1) [29–31]. Hence, using the genes obtained from our transcriptome dataset, a synthetic biology route was heterologous constructed to produce umbelliferone. Considering C4H expression in prokaryotes is difficult, RgTAL was selected to produce umbelliferone for it is able to bypass the hydroxylation step to produce *p*-coumaric acid (Fig. 1b) [32]. Eventually, *RgTAL, Ad4CL* and *AdC2’H* were cloned into prokaryotic expression vector according to the protocol for pCDF design in materials and methods part. As expected, a signal main peak was observed in the fermentation broth which was identified as umbelliferone (Fig. 3). However, we did not find any trace of *p*-coumaroyl-CoA, which is in accordance with most publications [32, 33]. The results indicated that 4CL may be the limiting factor for a high umbelliferone yield. In addition, the low yield and the requirement for additional L-tyrosine limited its industrial production. With the construction of a synthetic biology route for umbelliferone production accomplished, the next challenge would be to develop methods to improve the yield of umbelliferone using a raw carbon source.

### Construction of a tyrosine high-producing platform

**Fig. 4:**
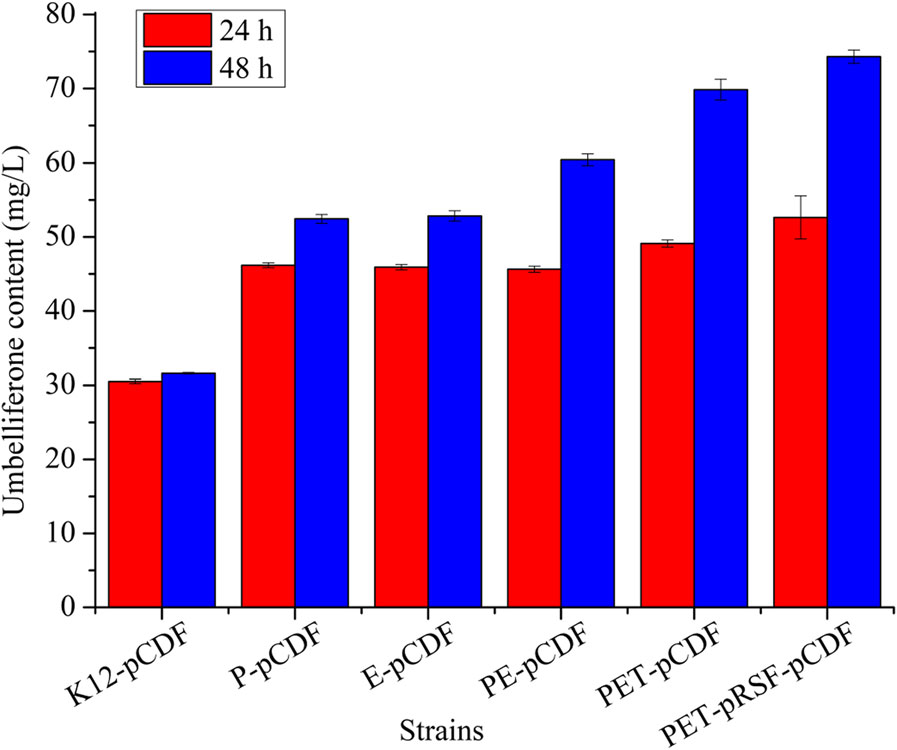
Comparison of different strains for the production of umbelliferone. Red and blue bars represent the umbelliferone concentration in 24 h and 48 h, respectively. The characteristics of different strains used in this experiment are listed in Table 3. All data are represented as mean ± SD from three independent experiments (*n* = 3). Error bars are defined as SD

To systematically improve the robustness of the synthetic pathway performance, a tyrosine high-producing platform was constructed. As depicted in Additional file 1: Figure S1, rational modular engineering included the 3-deoxy-arabinoheptulosonate-7-phosphate (DAHP) module, chorismate (CHA) module and tyrosine module. In the DAHP module, *transketolase* (*tktA*) was overexpressed to improve the metabolic flux of erythrose 4-phosphate (E-4-P), and the feedback resistant mutant *DAHP synthase* (*aroG*^*fbr*^) was simultaneously overexpressed to synthesize DAHP with E-4-P [34]. In CHA module, *dehydroquinate synthase*, *shikimate dehydrogenas*e, and *shikimate kinase I* (aroB, aroE and aroK) were placed in a single DNA fragment. However, each had an independent T7 and RBS sequences (pRSF) to improve the metabolic flux of DHAP into CHA [35, 36]. Studies have indicated that tyrR protein may suppress the expression of genes involved in aromatic amino-acid biosynthesis [37], and the *tyrR* was deleted from the genomic DNA of *E. coli.* In the tyrosine module, overexpressing the feedback-resistant mutant of (chorismate mutase/prephenate dehydrogenase) tyrA could enhance the accumulation of L-tyrosine, and knocking out *pheA* and *trpE* may reduce the loss of metabolic flux of CHA [34, 38]. Overexpression of *tyrA*^*fbr*^ and knock out of *pheA* and *trpE* was conducted to improve the potentially improve the high yield of tyrosine. Generally, after gene knockout and overexpression, six strains were obtained to analyze their ability in producing tyrosine (Table 2). The results indicate that a single knockout may not improve tyrosine yield. However, when *tyrR* was deleted, a clear improvement in the yield of tyrosine, approximately 6 times increase, was observed (Table 2). However, there was also a trend towards deleting genes that somewhat influenced bacterial growth, and a decreasing absorbance was observed. Hence, a triple knockout strain was used due to its high capacity for tyrosine synthesis (PET, Table 3). To analyze the ability of different strains to produce the umbelliferone, pCDF was introduced into the different strains. As indicated in Fig. 4, the strain knocking out *pheA, trpE*, *tyrR* and overexpressing pRSF (PET-pRSF) produced the highest umbelliferone, conforming to the tyrosine yield in Table 2.

**Table 2:** Effects of gene knockout and overexpress on the production of tyrosine

Strains	Growth (OD_600_/24 h)	L-tyrosine (mg/L)	Tyrosine/OD ratio
K12	1.86 ± 0.01	21.2 ± 0.1	11.3
P	1.41 ± 0.00	29.3 ± 0.1	20.8
E	1.39 ± 0.06	26.6 ± 0.1	19.1
PE	1.53 ± 0.03	61.1 ± 0.3	39.9
PET	1.08 ± 0.05	93.0 ± 0.2	86.1
PET-pRSF	1.16 ± 0.03	118.4 ± 0.4	102.1

**Table 3:** Strains and plasmids used and constructed in this study

	Description	Source
Strains		
*E.coli* DH5a	General cloning host	Invitrogen
*E. coli* K12 (MG1655)	F-, λ-, ilvG-, rfb-50, rph-1	Novagen
*E.coli* BL21(DE3)	F-, ompT, hsdS (r_B_^−^ m_B_^−^), gal, dcm (DE3)	Novagen
K12	*E. coli* K12 carrying the gene for *T7 RNA polymerase* in pGEX6P-1	This study
P	K12Δ*pheA*	This study
E	K12Δ*trpE*	This study
PE	K12Δ*pheAΔtrpE*	This study
PET	K12Δ*pheAΔtrpEΔtyrR*	This study
PET-pRSF	PET carrying pRSF	This study
K12-pCDF	K12 carrying pCDF	This study
P-pCDF	P carrying pCDF	This study
E-pCDF	E carrying pCDF	This study
PE-pCDF	PE carrying pCDF	This study
PET-pCDF	PET carrying pCDF	This study
PET-pRSF-pCDF	PET-pRSF carrying pCDF	This study
PET-pRSF-Pc4CL1	PET-pRSF carrying pCDF-Pc4CL1	This study
PET-pRSF-Pt4CL	PET-pRSF carrying pCDF-Pt4CL	This study
PET-pRSF-At4CL1	PET-pRSF carrying pCDF-At4CL1	This study
PET-pRSF-At4CL2	PET-pRSF carrying pCDF-At4CL2	This study
PET-pRSF-Pc4CL1-M	PET-pRSF carrying pCDF-Pc4CL1 (Q272H/F267 L)	This study
Plasmids		
pET28a	T7 promoters, pBR322 ori, Kn^R^	Novagen
pRSFDuet-1	Double T7 promoters, RSF ori, Kn^R^	Novagen
pCDFDuet-1	Double T7 promoters, CloDF13 ori, Sm^R^	Novagen
pRSF-1	pRSFDuet-1 carrying *tktA*	This study
pRSF-2	pRSFDuet-1 carrying *tktA*, *aroB*, *aroE* and *aroK*	This study
pRSF	pRSFDuet-1 carrying *tktA*, *aroG*^*fbr*^, *tyrA*^*fbr*^, *aroB*, *aroE* and *aroK*	This study
pCDF-1	pCDFDuet-1 carrying *RgTAL*	This study
pCDF-2	pCDFDuet-1 carrying *RgTAL, Ad4CL*	This study
pCDF	pCDFDuet-1 carrying *RgTAL, Ad4CL, AdC2’H*	This study
pCDF-Pc4CL1	pCDFDuet-1 carrying *RgTAL, Pc4CL1, AdC2’H*	This study
pCDF-Pt4CL	pCDFDuet-1 carrying *RgTAL, Pt4CL, AdC2’H*	This study
pCDF-At4CL1	pCDFDuet-1 carrying *RgTAL, At4CL1, AdC2’H*	This study
pCDF-At4CL2	pCDFDuet-1 carrying *RgTAL, At4CL2, AdC2’H*	This study

### Selection of enzyme sources and evolution of 4CL with protein engineering

**Fig. 5:**
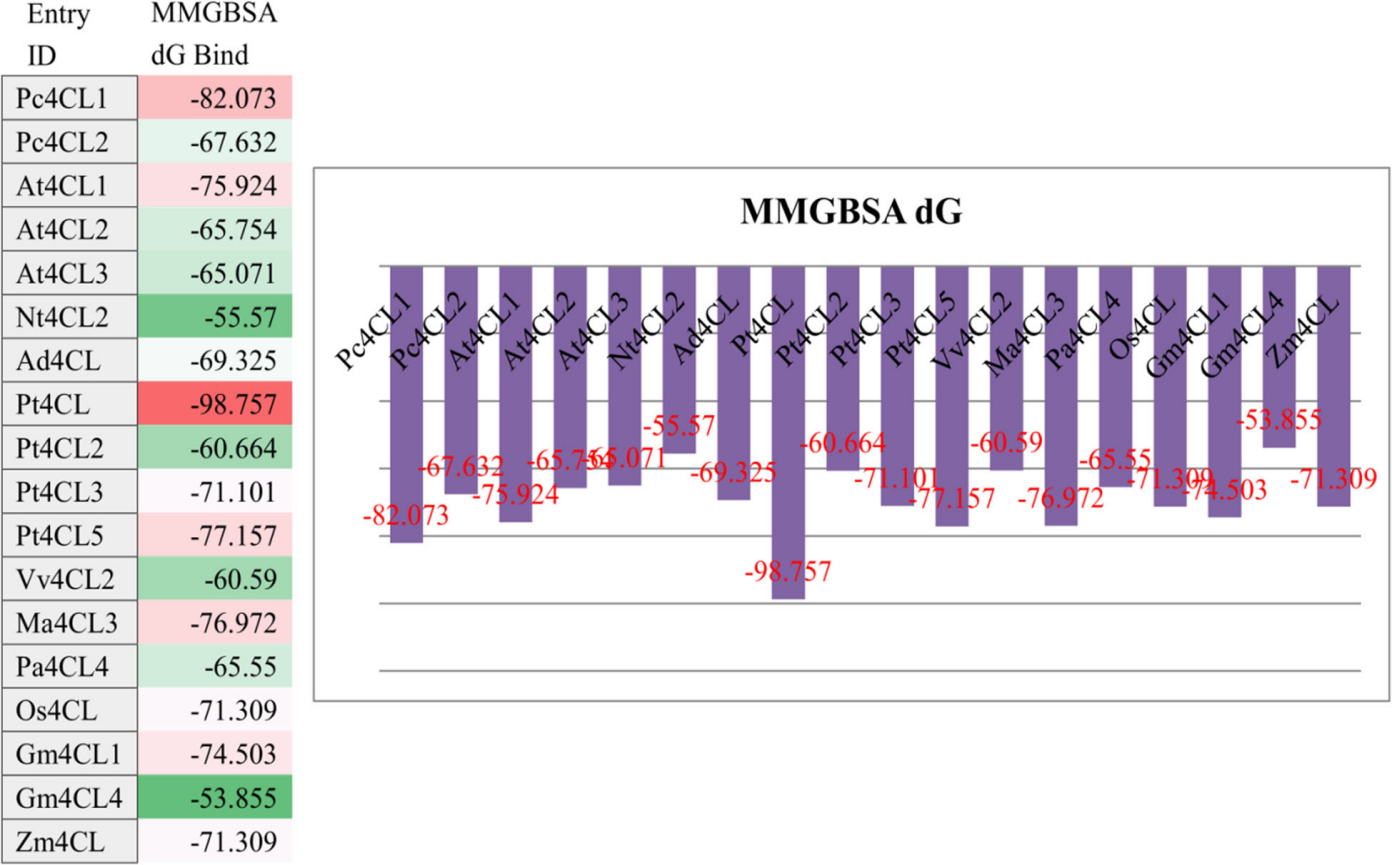
Virtual screening of different 4CLs using estimated binding energies (MM/GBSA dG) to adenosine 5′-coumaroyl phosphate. The different 4CLs estimated binding energies are listed on the left and are also drawn on the right

**Fig. 6:**
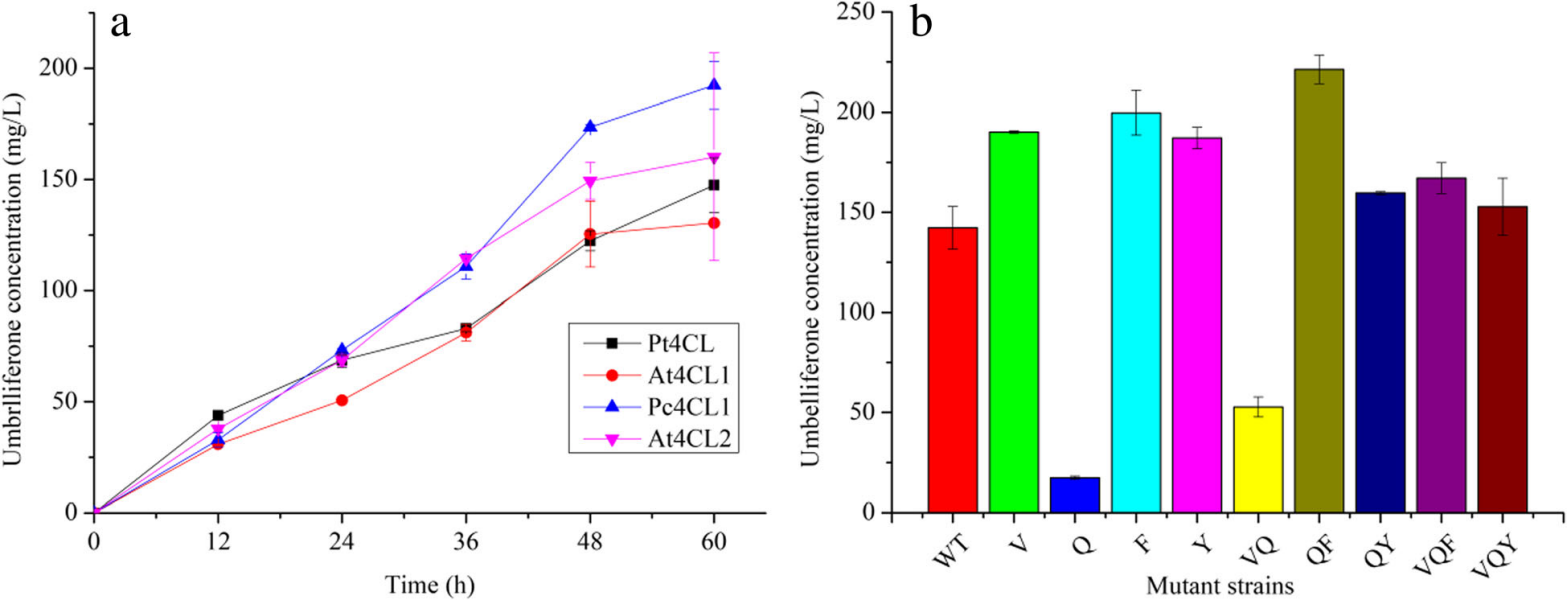
Umbelliferone production with different 4CLs and Pc4CL1 with different mutants. (**a**), The yield of umbelliferone in different strains expressing different 4CLs was measured for 60 h at 12 h increments. (**b**), The yield of umbelliferone with different Pc4CL1 mutations was measured at 48 h. V, Q, F, Y, VQ, QF, QY, VQF, VQY denotes Pc4CL1 V184G, Q272H, F267 L, Y240A, V184G/Q272H, Q272H/F267 L, Q272H/Y240A, V184G/Q272H/F267 L, V184G/Q272H/ Y240A mutations, respectively. All data are represented as mean ± SD from three independent experiments (*n* = 3). Error bars are defined as SD

In this study, a virtual screening strategy was used to select or optimize the enzyme with potential for product improvement. Based on the report that TAL from the red yeast, *R. glutinis,* showed the highest activity, compared with 7 other bacterial and fungal TAL enzymes, it was used to produce *p*-coumaric acid [39]. As 4CL activity appears to play a crucial role in compound production (Fig. 3), we next focused on how to select a suitable 4CL to convert *p*-coumaric acid. As there are only a few reports on 4CL selection, virtual screening and site-specific mutagenesis were used to select a candidate 4CL which efficiently produces umbelliferone. Firstly, eighteen 4CLs from different sources were virtually screened and sorted according to their estimated binding energies (Fig. 5). Then, four 4CLs showing potential for better.umbelliferone production were synthetized or PCR cloned, and used for expressing 4CL proteins. As indicated in Fig. 6a, 4CL1 from *P. crispum* tended to show a better performance in producing umbelliferone, despite ranking second compared with other 4CLs in our virtual screening. To further improve the yield of umbelliferone, a site-specific mutagenesis-based protein engineering approach was employed according to the directed evolution results of *Lycopersicon esculentum* 4CL (Le4CL) and the protein structure of Pt4CL [40, 41]. For instance, in Le4CL, V186G and F239S could improve the activity of Le4CL toward *p*-coumaric acid. Accordingly, the corresponding mutations of V184G, Q272H, F267 L, and so on, were generated in our target Pc4CL according to the sequence alignment results of Pc4CL, Le4CL and Pt4CL (Additional file 1: Figure S3). As indicated in Fig. 6b, the double mutant of Q272H and F267 L seemed play an important role in the good positive activity of 4CL. Hence, 4CL with Q272H and F267 L was used to produce compounds (PET-pRSF-Pc4CL1-M).

### Optimization of fermentation conditions to enhance umbelliferone production

Optimization of fermentation conditions was conducted to further enhance umbelliferone production. Considering that ions were also reported to affect the yield of gamma-aminobutyric acid [42], the effects of FeSO_4_, FeCl_3_, CaCl_2_, MgCl_2_, ZnSO_4_, CuSO_4_, MnSO_4_, CoCl_2_, NiCl_2_, LiAc and Na_2_MoO_4_ were investigated at concentration of 50, 100 and 500 mg/L were also investigated. As indicated in Additional file 1: Table S1, Mn^2+^ at a concentration of 100 mg/L positively enhanced the production of umbelliferone, where Co^2+^ played an inhibitory role. In addition, the inductor concentration (1, 10, 100, 1000 μM), induction temperature (15, 20, 25, 30, 35 °C) and induction time (5, 10, 15, 20, 25 h) were also investigated (Additional file 1: Table S2). Results indicated that while lactose at a concentration of 100 μM had the same effect as isopropyl β-D-thiogalactoside (IPTG), a low induction temperature and a high conversion temperature were favorable for enhancing the yield of products. Hence, ultimately, a conversion temperature of 40 °C, a lactose concentration of 100 μM and an induction temperature of 15 °C with an induction time of 5 h were used to further improve umbelliferone yield. Along with the selected strain and enzyme, an umbelliferone yield of 356.59 mg/L was obtained.

## Discussion

According to the compound structure identified from *A. decursiva,* we could partially estimate the biosynthetic mechanism of coumarin, despite the biosynthetic pathway of coumarins has not been completely investigated and no one gene has ever been reported in *A. decursiva* [3, 23]*.* As indicated in Fig. 1, at least four genes are involved in the biosynthesis of the coumarin skeleton. Considering that genes such as *PAL, C4H, 4CL* and *C2’H* from other species had been recently identified, we used the similarity search method to detect the corresponding candidate enzymes possibly involved in *A. decursiva* coumarin biosynthesis [24–26]. However, genomic data is unavailable at present. Due to NGS technology and annotation information available in NCBI, rapidity, and efficiency of gene discovery has improved dramatically [14, 15]. Hence, at the beginning of this study, we constructed the transcriptome dataset of *A. decursiva.* Next, a local BLAST search was conducted to predict candidate genes. As shown in Table 1, most genes have a high similarity to their template, indicating that candidate genes may serve the same function as known template genes. To confirm the genes functionally, we tested the function of all candidate genes in vitro, and the results indicated that all genes except C4H had corresponding functions (Fig. 2). Therefore, the umbelliferone biosynthesis in *A. decursiva* was established for the first time.

Considering that umbelliferone is an intermediate product of coumarin biosynthesis, an insufficient amount of umbelliferone can be accumulated in plants [23]. In addition, umbelliferone is a compound with biological activity and also acts as a precursor compound to produce various coumarin derivatives [31, 43, 44]. Hence, obtaining umbelliferone is urgent. Although many reports have been published on microbial production of secondary metabolites from glucose or mesostates, only a few focused on systematically improving target compound yields, and low yield makes these processes unfit for use under industrial conditions [16, 17, 45, 46]. In this study, we identified all genes involved in umbelliferone biosynthesis in *A. decursiva,* and re-structured its biosynthesis in vitro. The yields observed in our initial experiments were also low (Fig. 3). To improve candidate compound yield, a systematic strategy, consisting of integration of metabolic engineering and protein engineering, was conducted in engineered bacteria.

Considering that shikimate or tyrosine serves as the main precursor for the biosynthesis of phenylpropanoid compounds, we focused on the development of a microorganism platform with an enhanced capacity for shikimate/tyrosine production according to a previous report [34, 45]. Elimination of the bypass pathway and feedback inhibition was first conducted to reinforce tyrosine yield. Results indicated that simultaneous deletion of the tryptophan and phenylalanine bypass pathway could significantly enhance tyrosine yield (Table 2). Despite reports that overexpression of the genes involved in E-4-P, CHA and tyrosine biosynthesis may improve the final output of tyrosine, the effects were not as significant, in our experiment [34–36]. In contrast, deletion of *tyrR* from the genomic DNA of *E. coli* resulted in a considerable tyrosine yield. This phenomenon may be interpreted as being due to deletion of tyrR resulting in the inhibition of gene expression suppression involved in tyrosine biosynthesis [37]. Hence, a strain with null *pheA, trpE* and *tyrR* (PET) was used to produce tyrosine.

Development of precursor producing platforms to improve the yield of target compounds is somewhat effective, but it is often limited by the activity level of the downstream enzyme. A previous study has indicated that enzymes from different sources may affect the yield of products differently, and thus, selecting enzymes from different sources may provide an alternative way to further improve product yields. However, most researchers have focused on comparing enzymes from different sources, and little has been reported on improving enzyme performance in nature [19, 21]. In this study, a virtual screening strategy was conducted to select or optimize the enzymes with potential for product improvement. The work mainly focused on selecting a suitable 4CL to convert *p*-coumaric acid because *p*-coumaric acid can be copiously produced by RgTAL [39]. We first selected eighteen 4CLs for virtual screening using the mm/gbsa method by Prime (Prime, Schrödinger) [47]. Based on the estimated binding energies (Fig. 5), four 4CLs were selected to test for umbelliferone production ability. Based on the yield (Fig. 6), 4CL from *P. crispum* was used for site-specific mutagenesis according to the directed evolution results of Le4CL (Additional file 1: Figure S2) [40, 41]. The results indicated that double mutation of Q272H and F267 L may improve the yield of umbelliferone.

Actually, optimization of fermentation conditions could further improve the production of candidate compound despite there are little reports focused on this method [48]. Therefore, fermentation conditions together with induction conditions were optimized to further enhance umbelliferone yield. The effect of ions on the yield of umbelliferone was first investigated. Results indicated that Mn^2+^ may enhance umbelliferone production, which is in accordance with previous reports (Additional file 1: Table S1) [42]. Inductor concentration, induction time and induction temperature were also investigated (Additional file 1: Table S2). Considering economic factors, a conversion temperature of 4 °C, a lactose concentration of 100 μM, an induction temperature of 15 °C, and induction time of 5 h, are recommended for further improving umbelliferone yield. Although not much research has been conducted on optimization of fermentation conditions, our results indicated that optimizing fermentation conditions may significantly improve the yield of products.

## Conclusions

In this study, the RNA-seq dataset and the umbelliferone biosynthetic pathway in *A. decursiva were* elucidated for the first time. Furthermore, the genes involved in umbelliferone biosynthesis were introduced into microbial cells to produce umbelliferone. Metabolic as well as protein engineering was conducted to enhance umbelliferone yields. We used the gene knockout PET-pRSF strain, containing the Q272H and F267 L double mutation of Pc4CL1, to produce umbelliferone. Under optimized fermentation conditions, an umbelliferone yield of 356.59 mg/L was obtained. These findings proved that a combination of metabolic pathway optimization and protein engineering is useful in producing umbelliferone.

## Materials and methods

### Strains, plasmids and chemicals

Generally, *E. coli* DH5α was employed as the host for plasmid amplification and gene cloning. *E. coli* BL21 (DE3) and K12 (with T7-RNA polymerase) were used for recombinant protein expression, enzyme assays, and fermentation experiments. Unless otherwise stated, all strains and vectors were purchased from Invitrogen and Novagen and their characteristics are detailed in Table 3. Similarly, all chemicals were purchased from Sigma-Aldrich (St. Louis, MO, USA) or Aladdin (Shanghai, China). Antibiotics were purchased from Melonepharm (Dalian, China), unless otherwise indicated. Restriction enzymes and T4 DNA ligase were purchased from New England Biolabs (Hertfordshire, UK) or Takara (Dalian, China). Enzymes used for DNA amplification and kits used for RNA/plasmid/DNA isolation were purchased from Vazyme (Nangjing, China). The plasmids pKD3, pKD46 and pCP20 used for gene disruption were acquired from the Yale *E. coli* Genetic Stock Center.

### Plant materials, RNA isolation, library preparation, sequencing, assembly and functional annotation

*A. decursiva* material was collected from our medicinal botanical garden at China Pharmaceutical University. The plant was immediately frozen in liquid nitrogen and stored at − 80 °C until use. Total RNA was isolated using TransZol Plant reagent (TransGen Biotech, Beijing, China) according to the user guidelines. After integrity and quality checks, the RNA was used as a template for cDNA amplification using SMARTerTM RACE Amplification Kit (Clontech Laboratories, Inc., Mountain View, CA, USA) and TruSeq Stranded mRNA Library Prep Kit (Illumina). Subsequently, the cDNA was subjected to end-repair, phosphorylation and “A” base addition according to the library construction protocol. Sequencing, assembly and functional annotation methods were according to our previous report [23]. The RNA-seq data generated in this work have been deposited in the SRA (Sequence Read Archive) database under BioProject ID PRJNA360870.

### Characterization of genes in the umbelliferone pathway and cDNA cloning

To identify the target nucleic acid sequences, a local BLAST search was conducted using the program of TBLASTN in Bioedit Sequence Alignment Editor according to the deep sequencing dataset of *A. decursiva.* For details, previously reported functionally identified protein sequences were extracted from NCBI and used for template for local homologous BLAST searches [25–27]. The template used in this work, the local BLAST results for each gene and their E-Values are listed in Table 1. Unigenes exhibiting the highest similarity were used to design primers for amplifying full-length cDNA from *A. decursiva* using SMARTer™ RACE Amplification Kit [23]. The PCR products were cloned into the pMD19-T vector (Takara, Dalian, China) for DNA sequencing. After sequencing, the PCR fragments were joined reveal the open reading frames (ORFs). Finally, the candidates were re-amplified using gene-specific primers with the corresponding primers for construction of the expression vectors. All the primers used in this work are listed in Additional file 1: Table S3.

### Protein expression, purification and enzymatic reaction

All of the genes were cloned into pET28a with an N-terminal fusion histidine tag for expression and purification. Recombinant plasmids were first introduced into *E. coli* BL21 (DE3), and the bacteria were cultured in 200 mL Luria-Bertani medium at 37 °C until the OD 600 reached 0.4–0.8. 100 μM IPTG was added followed by overnight induction at 16 °C. For protein purification, the culture was centrifuged at 5000×g for 10 min at 4 °C. Then, the cells were re-suspended in buffer (50 mM NaH_2_PO_4_, 300 mM NaCl, pH 8.0) and centrifuged again. After ultrasonication, the re-suspended cells were centrifuged at 15000×g for 30 min at 4 °C to remove the cell debris. The supernatant was used for protein purification with a Ni-NTA affinity column and FPLC (ÄKTA, GE Healthcare Bio-Sciences) according to our previous study [25]. Finally, protein concentrations were determined using the Bradford kit (Jiancheng, Nanjing, China) according to the technical manual, and the samples were was stored at − 80 °C until use. For enzymatic reactions, different proteins (1 μg) were incubated with various substrates (approximately 1 mM) at 37 °C for 30 min (100 mM Tris-HCl pH 8.0). When necessary, 5 mM ATP, 5 mM MgC1_2_, and 0.3 mM CoA were added to the reaction system according to our previous reports [25, 26].

### Reconstruction of the metabolic pathway

According to the metabolic pathway involved in the biosynthesis of the umbelliferone, gene knockout was first employed to optimize the metabolic route to construct a high yield platform using the classical λ Red homologous recombination method [49]. Specifically, *pheA, trpE, tyrR* were knocked out, singularly or in combination, to produce tyrosine (Table 3) [34]. All primers used in this work are listed in Additional file 1: Table S3.

### Construction of expression vectors

The plasmid used for precursor supply was constructed in pRSFDuet-1. First, six genes (*tktA*, *aroG*, *tyrA*, *aroB*, *aroE*, *aroK*) were PCR amplified and cloned into PMD19-T for overlap or site-specific mutagenesis. The genes were constructed into pRSFDuet-1 by T4 DNA ligase or overlap PCR when necessary. *TktA* was cloned into pRSFDuet-1 directly between 5′-Nco I and Sac I-3′ sites to produce pRSFDuet-*tktA* (pRSF-1). The *aroE* and *aroK* first pieced together with the T7 and RBS sequences to generate T7-RBS-*aroE* and T7-RBS*-aroK* and then overlapped with *aroB* to generate *aroB*-T7-RBS-*aroE* -T7-RBS*-aroK*. The overlapping fragment was ligated to pRSF-1 to generate pRSFDuet-*tktA*-*aroB-aroE-aroK* (pRSF-2) with 5′-EcoR V and Avr II-3′. For *aroG* and *tyrA* construction, the genes were first mutated to generate feedback-inhibition-resistant (fbr) genes *aroG*^*fbr*^ and *tyrA*^*fbr*^ using KOD-plus-neo (Toyobo, Toyobo Life Science Department). Subsequently, aroG^fbr^ and tyrA^fbr^ were joined with T7 and RBS sequences to generate T7-RBS-*aroG*^*fbr*^-T7-RBS-*tyrA*^*fbr*^, and the fragment was ligated to pRSF-2 to yield pRSFDuet-*tktA*-*aroG*^*fbr*^-*tyrA*^*fbr*^*-aroB-aroE-aroK* (pRSF) with 5′-Sac I and Not I-3′.

The compound synthetic pathway consists of three enzymes: RgTAL, Ad4CL and AdC2’H. As it described above, TAL and 4CL were directly synthesized and cloned into the multi-cloning site of pCDFDuet-1 at 5′-Nco I/BamH I-3′ and 5′-Nde I/Kpn I-3′ to yield pCDFDuet-TAL and pCDFDuet-TAL-4CL (pCDF-1 and pCDF-2), respectively. Similarly, C2’H was inserted into the second multi-cloning site of pCDF-2 at 5′- Kpn I/Xho I-3′ (pCDF). All primers used to construct expression vectors are listed in Additional file 1: Table S3.

### Virtual screening and site-specific mutagenesis of 4CL

To select a candidate 4CL for efficiently producing umbelliferone, different sources of 4CL were first virtually screened and then further mutated by site-specific mutagenesis. Approximately 18 4CLs from different resources were first selected (Additional file 1: Table S4), and, all structures except for *Arabidopsis thaliana* 4CL1 (At4CL1) and Pt4CL were predicted using SWISS-MODEL [50] because the crystal structures of At4CL1 and Pt4CL have been resolved and are available in the Protein Data Bank (PDB, 3TSY and 3NI2/3A9V) [41, 51]. The structures of adenosine 5′-coumaroyl phosphate and proteins were prepared with the LigPrep module and Protein Preparation Wizard module (Schrödinger 2014, LLC, New York, NY) [52]. The core pattern was adopted considering that adenosine 5′-coumaroyl phosphate is somewhat large and contains many rotatable bonds. That is, the binding mode of 3NI2 (Pt4CL and adenosine 5′-(3-(4-hydroxyphenyl) propyl) phosphate) was used as a reference for docking adenosine 5′-coumaroyl phosphate to all 18 proteins (Additional file 1: Table S4). As the structural differences among these enzymes were also taken into account, a rather large tolerance value of 5 Å was adopted when docking. Docking was carried out using the Glide module (Glide, Schrödinger) and the results were selected and refined by the Refine Protein-Ligand Complex program in the Prime module (Prime, Schrödinger) (Additional file 1: Figure S2) [47]. Binding energies were estimated through the mm/gbsa method in Prime, and the 18 enzymes were sorted according to estimated binding energies. Finally, the candidate 4CL was cloned into pCDF-1 for fermentation. To further improve the yield of the umbelliferone, site-specific mutagenesis was conducted to produce a series of mutants. Firstly, according to the protein structure of Pt4CL and the directed evolution results of Le4CL, key amino acid residues which may play a better performance in 4CL activity was selected [40, 41]. For instance, V186, F239, Q274 in Le4CL and Y236 in Pt4CL. Then sequence alignment was conducted among Pc4CL1, Le4CL and Pt4CL to find the amino acid residues in Pc4CL corresponding to V186, F239, Q274 in Le4CL and Y236 in Pt4CL [40, 41]. Multiple sequence alignment was performed using DNAMAN (Lynnon Corp., Pointe-Claire, QC, Canada) and the protein accession numbers used in sequence alignment are X13324.1, NP_001333770.1 and AY043495.1 for Pc4CL1, Le4CL and Pt4CL, respectively (Additional file 1: Figure S3) [40, 41]. At last, candidate mutations were generated according to the results in Le4CL and Pt4CL. For instance, in *Le*4CL, V186G and F239S could improve the activity of Le4CL towards *p*-coumaric acid. In our target Pc4CL, the corresponding mutations of V184G, Q272H, F267 L, and so on, were generated. All primers used are listed in Additional file 1: Table S3.

### Culture and biotransformation conditions and experimental design

For recombinant plasmid construction, gene knockout and seed culture, LB medium (10 g/L tryptone, 5 g/L yeast extract and 10 g/L NaCl) with various concentrations of antibiotics (ampicillin 100 mg/L, chloramphenicol 34 mg/L, kanamycin 50 mg/L and streptomycin 50 mg/L) as used. For shake flask fermentation, the seed culture was incubated overnight in LB medium, and then 0.25 mL of the seeds were inoculated into a triangle flask containing 25 mL medium. FeSO_4_, FeCl_3_, CaCl_2_, MgCl_2_, ZnSO_4_, CuSO_4_, MnSO_4_, CoCl_2_, NiCl_2_, LiAc and Na_2_MoO_4_ at concentrations of 50, 100 and 500 mg/L were added to investigate the effects of ions on product yield. In addition, the concentration (IPTG and lactose at concentrations of 1, 10, 100, 1000 μM), temperature (15, 20, 25, 30, 35 °C) and time (5, 10, 15, 20, 25 h) of induction were investigated. All medium and fermentation conditions were optimized using M9 minimal medium with an induction temperature of 16 °C and a transformation temperature of 35 °C.

### High performance liquid chromatography (HPLC) and yield analysis

Reversed phase HPLC using C18 column (XDB-C18, 5 mm; Agilent, USA) was conducted to analyze candidate compounds at a flow of 1 mL/min. For sample preparation, the fermentation broth was collected by centrifugation and the supernatant was then injected for analysis. For PAL and C4H analysis, 42% acetonitrile isocratic was used for approximately 15 min. For 4CL and C2’H analysis, the solvent gradient conditions A (H_2_O): B (methanol; v/v) were as follows: 0 min, 95:5; 5 min, 90:10; 15 min, 40:60; 20 min, 10:90. For umbelliferone analysis, the solvent gradient conditions A (H_2_O): B (methanol; v/v) were as follows: 0 min, 95:5; 5 min, 40:60; 15 min, 5:95.

## Additional file

Additional file 1: **Figure S1** Experimental design of construction of a tyrosine high-producing platform. The overexpressed genes in this work are marked with red arrow and the knocked genes are marked with brown. TAL, tyrosine ammonia lyase; 4CL, 4-coumarate: coenzyme A ligase; C2'H, *p*-coumaroyl CoA 2'-hydroxylase; E-4-P, erythrose 4-phosphate; CHA, chorismate; DAHP, 3-deoxy-arabinoheptulosonate-7-phosphate; tktA, transketolase; aroG^fbr^, feedback resistant mutant DAHP synthase; tyrA^fbr^, feedback resistant mutant chorismate mutase/prephenate dehydrogenase ; aroB, dehydroquinate synthase; aroE, shikimate dehydrogenase; aroK, shikimate kinase I; tyrR, transcriptional regulatory protein; pheA, prephenate dehydratase; trpE, anthranilate synthase. **Figure S2** Homology modeling and docking of Pc4CL with adenosine 5’-coumaroyl phosphate. The amino acid main chains are displayed in ribbon and the main amino acids are marked with globular and stick. **Figure S3** Sequence alignment of Pc4CL with Le4CL and Pt4CL. Multiple sequence alignment was performed using DNAMAN and the protein accession numbers used in sequence alignment are X13324.1, NP_001333770.1 and AY043495.1 for Pc4CL1, Le4CL and Pt4CL, respectively. The selected mutation sites are marked with black squares. **Table S1** Effects of different ions on the production of umbelliferone. **Table S2** Effects of fermentation conditions on the production of umbelliferone. **Table S3** Primers used in this study. **Table S4** Candidate 4CLs used for virtual screening. (DOCX 2084 kb)
