# Supplementary material for: Elucidation of the biosynthesis pathway and heterologous construction of a sustainable route for producing umbelliferone
Source: J Biol Eng. 2019 May 22;13:44. doi: 10.1186/s13036-019-0174-3 (PMC6530170; doi:10.1186/s13036-019-0174-3)
Supplement: Supplementary file 1 — Figure S1 Experimental design of construction of a tyrosine high-producing platform. The overexpressed genes in this work are marked with red arrow and the knocked genes are marked with brown. TAL, tyrosine ammonia lyase; 4CL, 4-coumarate: coenzyme A ligase; C2'H, p-coumaroyl CoA 2'-hydroxylase; E-4-P, erythrose 4-phosphate; CHA, chorismate; DAHP, 3-deoxy-arabinoheptulosonate-7-phosphate; tktA, transketolase; aroGfbr, feedback resistant mutant DAHP synthase; tyrAfbr, feedback resistant mutant chorismate mutase/prephenate dehydrogenase ; aroB, dehydroquinate synthase; aroE, shikimate dehydrogenase; aroK, shikimate kinase I; tyrR, transcriptional regulatory protein; pheA, prephenate dehydratase; trpE, anthranilate synthase. Figure S2 Homology modeling and docking of Pc4CL with adenosine 5’-coumaroyl phosphate. The amino acid main chains are displayed in ribbon and the main amino acids are marked with globular and stick. Figure S3 Sequence alignment of Pc4CL with Le4CL and Pt4CL. Multiple sequence alignment was performed using DNAMAN and the protein accession numbers used in sequence alignment are X13324.1, NP_001333770.1 and AY043495.1 for Pc4CL1, Le4CL and Pt4CL, respectively. The selected mutation sites are marked with black squares. Table S1 Effects of different ions on the production of umbelliferone. Table S2 Effects of fermentation conditions on the production of umbelliferone. Table S3 Primers used in this study. Table S4 Candidate 4CLs used for virtual screening. (DOCX 2084 kb) [file 13036_2019_174_MOESM1_ESM.docx]

**Supplementary Information**

**Elucidation of the biosynthesis pathway and heterologous construction of a sustainable route for producing umbelliferone**

Yucheng Zhao, Xiangyun Jian, Jialin Wu, Wanchun Huang, Chuanlong Huang, Jun Luo* and Lingyi Kong*

Jiangsu Key Laboratory of Bioactive Natural Product Research and State Key Laboratory of Natural Medicines, School of Traditional Chinese Pharmacy, China Pharmaceutical University, Nanjing 210009, People's Republic of China


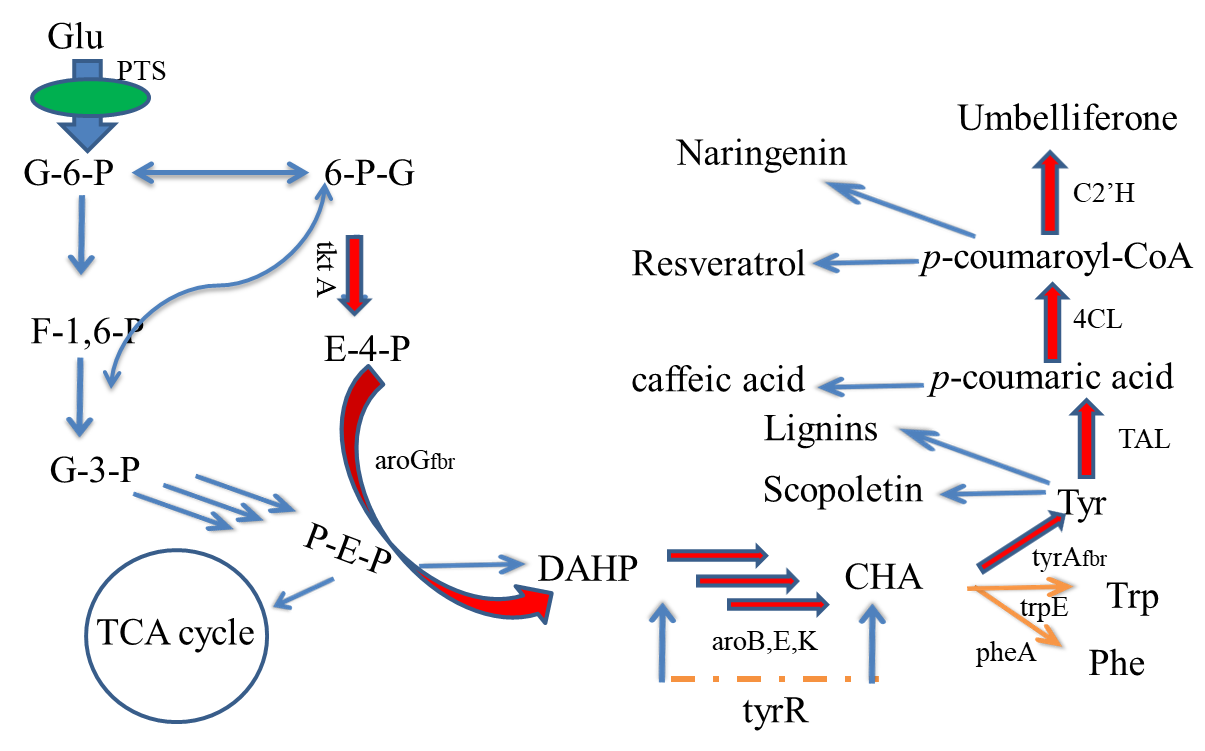


**Fig. S1** Experimental design of construction of a tyrosine high-producing platform. The overexpressed genes in this work are marked with red arrow and the knocked genes are marked with brown. TAL, tyrosine ammonia lyase; 4CL, 4-coumarate: coenzyme A ligase; C2'H, [*p*-coumaroyl CoA 2'-hydroxylase](https://www.ncbi.nlm.nih.gov/protein/ASR80916.1/); E-4-P, erythrose 4-phosphate; CHA, chorismate; DAHP, 3-deoxy-arabinoheptulosonate-7-phosphate; tktA, transketolase; aroG^fbr^, feedback resistant mutant DAHP synthase; tyrA^fbr^, feedback resistant mutant chorismate mutase/prephenate dehydrogenase ; aroB, dehydroquinate synthase; aroE, shikimate dehydrogenase; aroK, shikimate kinase I; tyrR, transcriptional regulatory protein; pheA, prephenate dehydratase; trpE, anthranilate synthase.


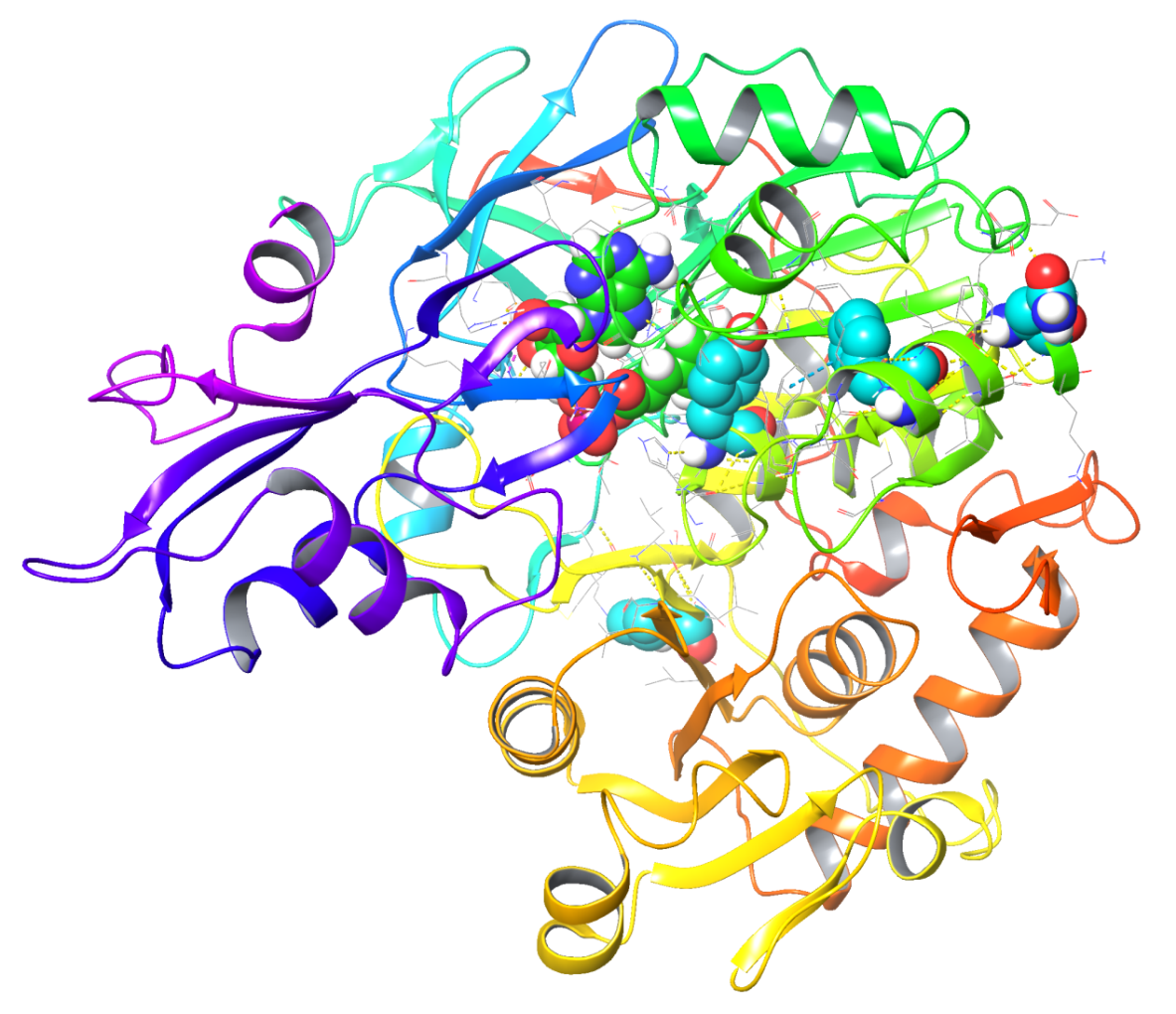


**Fig. S2** Homology modeling and docking of Pc4CL with adenosine 5’-coumaroyl phosphate.

The amino acid main chains are displayed in ribbon and the main amino acids are marked with globular and stick.


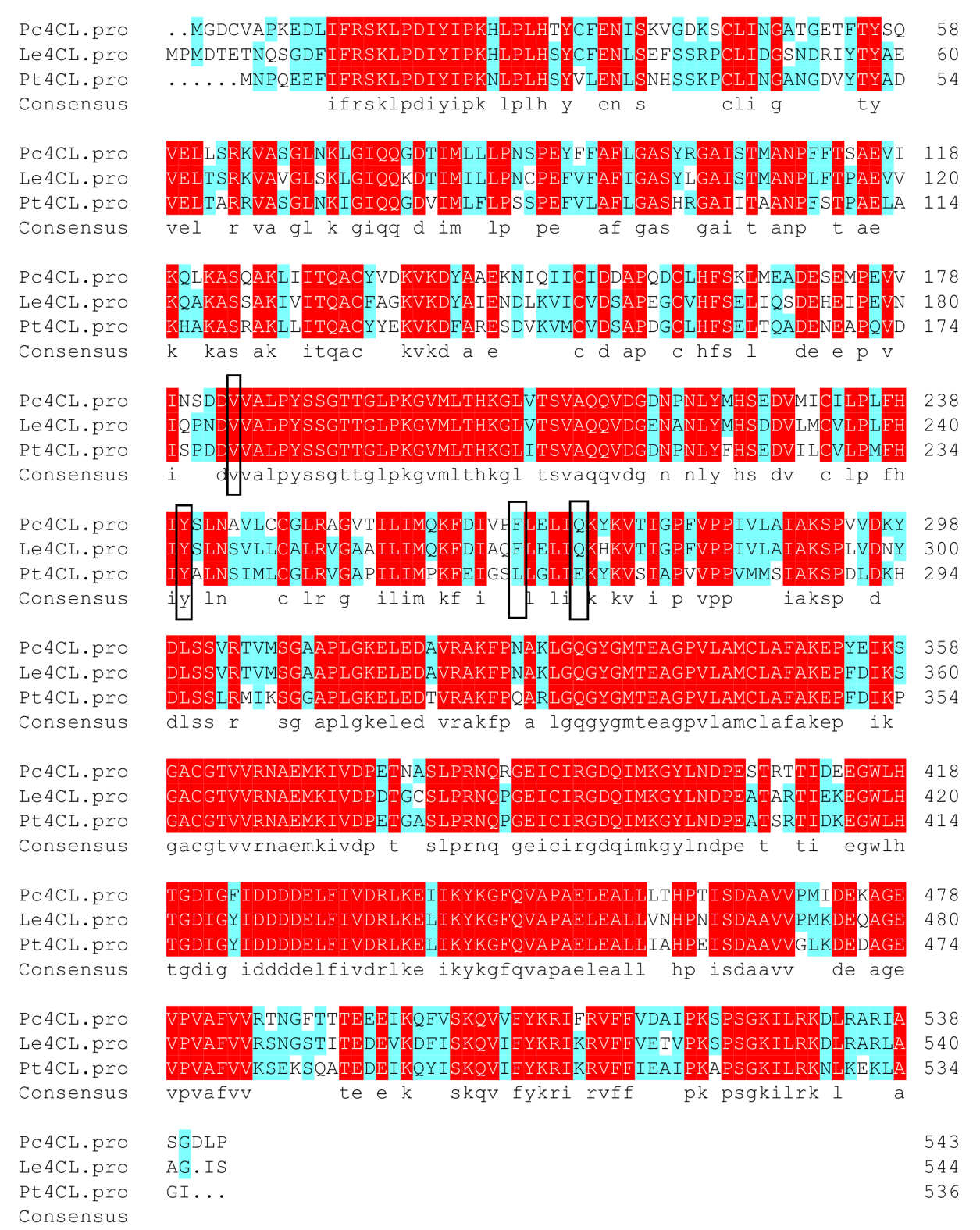


**Fig. S3** Sequence alignment of Pc4CL with Le4CL and Pt4CL. Multiple sequence alignment was performed using DNAMAN and the protein accession numbers used in sequence alignment are X13324.1, NP_001333770.1 and AY043495.1 for Pc4CL1, Le4CL and Pt4CL, respectively. The selected mutation sites are marked with black squares.

**Table S1** Effects of different ions on the production of umbelliferone.

|  | | **Umbelliferone** | |
| --- | --- | --- | --- |
|  |  | **24h** | **48h** |
| **Ca^2+^** | 50 mg/L | 140.62±31.58 | 195.21±9.05 |
|  | 100 mg/L | 162.02±8.29 | 191.81±5.18 |
|  | 150 mg/L | 148.29±18.47 | 176.41±24.69 |
| **Co^2+^** | 50 mg/L | 131.69±13.29 | 191.08±32.80 |
|  | 100 mg/L | 112.03±32.23 | 157.75±19.79 |
|  | 150 mg/L | 27.52±0.57 | 27.12±0.00 |
| **Cu^2+^** | 50 mg/L | 151.82±8.39 | 196.01±15.83 |
|  | 100 mg/L | 146.76±20.26 | 185.55±19.89 |
|  | 150 mg/L | 111.3±4.62 | 158.09±7.26 |
| **Fe^2+^** | 50 mg/L | 148.09±5.37 | 203.21±17.53 |
|  | 100 mg/L | 147.89±18.85 | 174.81±14.89 |
|  | 150 mg/L | 127.23±10.37 | 180.28±9.99 |
| **Fe^3+^** | 50 mg/L | 155.95±18.95 | 181.81±12.16 |
|  | 100 mg/L | 153.89±18.29 | 191.34±17.53 |
|  | 150 mg/L | 129.89±11.88 | 167.28±9.14 |
| **Li^2+^** | 50 mg/L | 161.29±12.16 | 187.61±19.04 |
|  | 100 mg/L | 121.1±17.15 | 187.88±3.58 |
|  | 150 mg/L | 158.62±7.63 | 203.34±0.01 |
| **Mg^2+^** | 50 mg/L | 158.22±27.24 | 202.41±0.94 |
|  | 100 mg/L | 136.03±8.48 | 185.28±0.28 |
|  | 150 mg/L | 144.09±29.88 | 179.75±5.09 |
| **Mn^2+^** | 50 mg/L | 166.55±11.50 | 233.4±10.09 |
|  | 100 mg/L | 161.29±23.66 | 244.46±2.17 |
|  | 150 mg/L | 163.88±24.32 | 214.94±49.01 |
| **MoO_4_^2-^** | 50 mg/L | 155.62±11.31 | 204.94±0.32 |
|  | 100 mg/L | 141.09±10.74 | 192.68±2.54 |
|  | 150 mg/L | 146.16±20.17 | 210.94±3.16 |
| **Ni^2+^** | 50 mg/L | 149.36±5.47 | 191.54±0.85 |
|  | 100 mg/L | 156.82±3.20 | 198.07±5.00 |
|  | 150 mg/L | 108.23±16.49 | 184.41±22.43 |
| **Zn^2+^** | 50 mg/L | 153.95±18.00 | 186.01±13.57 |
|  | 100 mg/L | 153.35±29.41 | 185.75±13.01 |
|  | 150 mg/L | 47.92±29.41 | 103.17±95.48 |

**Table S2** Effects of fermentation conditions on the production of umbelliferone.

|  | | **Umbelliferone** | |
| --- | --- | --- | --- |
|  |  | **24h** | **48h** |
| **Conversion temperature** | 25℃ | 84.91±5.66 | 84.11±8.77 |
|  | 30℃ | 115.33±8.25 | 120.96±5.37 |
|  | 35℃ | 158.42±10.56 | 199.54±16.02 |
|  | 40℃ | 216.37±18.05 | 251.36±40.67 |
| **IPTG concentration** | 10 μM | 190.21±11.5 | 240.06±3.02 |
|  | 100 μM | 149.59±1.18 | 198.51±37.94 |
|  | 250 μM | 144.89±17.53 | 223.60±2.260 |
|  | 500 μM | 162.95±8.67 | 209.97±12.21 |
|  | 1000 μM | 163.52±22.29 | 286.15±66.59 |
| **Lactose concentration** | 10 μM | 86.31±2.26 | 139.29±1.60 |
|  | 100 μM | 132.43±29.22 | 250.46±55.14 |
|  | 1000 μM | 179.81±7.45 | 242.00±8.48 |
|  | 2000 μM | 193.54±3.49 | 232.93±4.90 |
| **Induction time** | 5 h | 122.08±9.52 | 190.01±11.31 |
|  | 10 h | 183.64±3.91 | 192.08±39.68 |
|  | 15 h | 193.09±2.51 | 199.54±16.02 |
| **Induction temperature** | 15℃ | 119.42±7.31 | 199.54±16.02 |
|  | 20℃ | 61.07±5.24 | 101.90±15.65 |
|  | 25℃ | 43.92±1.53 | 113.90±4.95 |

**Table S3** Primers used in this study.

| Primer name | (5' to 3') | | |
| --- | --- | --- | --- |
| AdPAL-F | GTGCCGCGCGGCAGCCATATGGAGTGCGATAATGGTGCA | | |
| AdPAL-R | TGTCGACGGAGCTCGAATTCTTAACAGATTGGAAGAGGAG | | |
| AdC4H-F | GTGCCGCGCGGCAGCCATATGGATTTTCTGTTCTTGGAG | | |
| AdC4H-R | TTGTCGACGGAGCTCGAATTCTTATAATGATCTTGGCTTGCAG | | |
| Ad4CL-F | GTGCCGCGCGGCAGCCATATGGGAGATTGTGCAGCACC | | |
| Ad4CL-R | TTGTCGACGGAGCTCGAATTCTTATTTGGGAAGATCACCGG | | |
| AdC2’H-F | GTGCCGCGCGGCAGCCATGCTCCATCTACCACTGATGAT | | |
| AdC2’H-R | TGTCGACGGAGCTCGAATTCTCACATCTTTGCGAAATCAAGAG | | |
| pheA-F | CCCAAATCGGGGGGCCTTTTTTATTGATAACAAAAAGGCAACACTATGGTGTAGGCTGGAGCTGCTTC | | |
| pheA-R | GATTCACATCATCCGGCACCTTTTCATCAGGTTGGATCAACAGGCACCATGGGAATTAGCCATGGTCC | | |
| pheA-chk-F | GTCGTGTGAAACAGAATGCG | | |
| pheA-chk-R | TGCGTCAGGCGAATGACAAT | | |
| trpE-F | CCCGCCTAATGAGCGGGCTTTTTTTTGAACAAAATTAGAGAATAACAATGGTGTAGGCTGGAGCTGC | | |
| trpE-R | GTTGTACGTAAAAGAGTCGATATTATCGAGCAGCAGAATGTCAGCCATCAATGGGAATTAGCCATGG | | |
| trpE-chk-F | ACCCAGCCCGCCTAATGAGC | | |
| trpE-chk-R | CGCAACTGATCTGCCAGGTT | | |
| tyrR-F | GTGTCATATCATCATATTAATTGTTCTTTTTTCAGGTGAAGGTTCCCATGGTGTAGGCTGGAGCTGC | | |
| tyrR-R | ATAATTTAATATGCCTGATGGTGTTGCACCATCAGGCATATTCGCGCTTAATGGGAATTAGCCATGG | | |
| tyrR-chk-F | GAAGTGCCCGTTTTTCCGTC | | |
| tyrR-chk-R | CGCGTGCCGTTGTGGTTATT | | |
| Nco I-accBC-F | CATGCCATGGCCGTGTCAGTCGAGACTAGG | | |
| accBC-BamH I-R | CGGGATCCTTACTTGATCTCGAGGAGAACA | | |
| EcoR V-dtsR1-F | GCCGATATCCATGACCATTTCCTCACCT | | |
| dtsR1-Kpn I-R | GGGGTACCTTACAGTGGCATGTTGCCGT | | |
| PTDH-T7-RBS-F | CCCAAGCTTGCGACTCCTGCATTAGGAAAT | | |
| Sse-T7-tyrA-F  T7-tyrA-NotI-R  Kpn I-T7-aroE-F  T7-aroE-Xho I-R  Xho I-T7-aroK-F  T7-aroK-AvrII-R  T7-RNA-EcoN I-F  T7-RNA-EcoR I-R | TATACCTGCAGGATGCGTCCGGCGTAGA  ATAGTTTAGCGGCCGCTTACTGGCGATT  GATAGGTACCTTGTACACGGCCGCATAATC  AGTAACTCGAGTCACGCGGACAATTCCTCC  TATACTCGAGTTGTACACGGCCGCATAATC  CCTACCTAGGTTAGTTGCTTTCCAGCATGT  CCTATACTAGGTATGAACACGATTAACATCGC  GAATTCTTACGCGAACGCGAAGTCC | | |
| BamH I-T7RNA-F | | CGCGGATCCATGAACACGATTAACATCG |  |
| T7RNA-EcoR I-R | | TTAGAATTCCGCGAACGCGAAGTCCGAC |  |
| pGEX-chk-F  pGEX-chk-R  Nco I-RgTAL-F  RgTAL-BamH I-R  Pt4CL-Nde I-F  Pt4CL-Kpn I-R  Pt4CL-M-Xho-1R  Pt4CL-M-Xho-2F  Pc4CL1-Nde I-F  Pc4CL1- Kpn I-R  At4CL1-Nde I-F  At4CL1-Kpn I-R  At4CL2-Nde I-F  At4CL2-Kpn I-R  Ad4CL-Nde I-F  Ad4CL- Kpn I-R  AdC2’H-Kpn I-F  AdC2’H-Xho I-R  V184G-F  V184G-R  Q272H-F  Q272H-R  F267L-F  F267L-R  Y240A-F  Y240A-R | | GGGCTGGCAAGCCACGTTTGGTG  CCGGGAGCTGCATGTGTCAGAGG  CCATGGCACCGCGCCCGACCAGCCAGAGCC  GGATCCCGCCAGCATTTTCAGCAGCACGTT  CGGCCATATGAATCCACAAGAAGAATTC  GGGGTACCTTATATGCCTGCCAACTTTT  CAGAAGCTTTGCTCTGGAGGCCTTGGCATG  CATGCCAAGGCCTCCAGAGCAAAGCTTCTG  CATATGGGCGATTGCGTGGCCCCGAAAGAA  GGTACCTTATTTCGGCAGATCGCCGCTGGC  CATATGGCGCCACAAGAACAA  GGTACCTCACAATCCATTTGCTAG  CATATGACGACACAAGATGTG  GGTACCCTAGTTCATTAATCCATTTGC  CATATGGAAAAATCCGGTTATGG  GGTACCTTATTTGGGAAGATCACCGGATGC  GGATCCTGCATTAGGAAATTAATACGACTC  CTCGAGTTATTCAATGCGCGCAAAC  ATTGATAGCGATGATGGGGTGGCGCTGCCGTAC  GTACGGCAGCGCCACCCCATCATCGCTATCAAT  TCTGGAACTGATTCACAAATATAAAGTGAC  GTCACTTTATATTTGTGAATCAGTTCCAGA  TGATATTGTGCCGCTTCTGGAACTGATTC  GAATCAGTTCCAGAAGCGGCACAATATCA  GCTGTTTCACATTGCCAGCCTGAATGCCGTGC  GCACGGCATTCAGGCTGGCAATGTGAAACAGC |  |
|  |  | | |

**Table S4** Candidate 4CLs used for [virtual screening](http://dict.youdao.com/search?q=virtual%20screening%0D%0A&keyfrom=fanyi.smartResult).

| Protein name/  Accession number | Amino acid sequences | | |
| --- | --- | --- | --- |
| [*P. crispum*](https://www.ncbi.nlm.nih.gov/Taxonomy/Browser/wwwtax.cgi?id=4043) Pc4CL1  X13324.1 | MGDCVAPKEDLIFRSKLPDIYIPKHLPLHTYCFENISKVGDKSCLINGATGETFTYSQVELLSRKVASGLNKLGIQQGDTIMLLLPNSPEYFFAFLGASYRGAISTMANPFFTSAEVIKQLKASQAKLIITQACYVDKVKDYAAEKNIQIICIDDAPQDCLHFSKLMEADESEMPEVVINSDDVVALPYSSGTTGLPKGVMLTHKGLVTSVAQQVDGDNPNLYMHSEDVMICILPLFHIYSLNAVLCCGLRAGVTILIMQKFDIVPFLELIQKYKVTIGPFVPPIVLAIAKSPVVDKYDLSSVRTVMSGAAPLGKELEDAVRAKFPNAKLGQGYGMTEAGPVLAMCLAFAKEPYEIKSGACGTVVRNAEMKIVDPETNASLPRNQRGEICIRGDQIMKGYLNDPESTRTTIDEEGWLHTGDIGFIDDDDELFIVDRLKEIIKYKGFQVAPAELEALLLTHPTISDAAVVPMIDEKAGEVPVAFVVRTNGFTTTEEEIKQFVSKQVVFYKRIFRVFFVDAIPKSPSGKILRKDLRARIASGDLPK | | |
| [*P. crispum*](https://www.ncbi.nlm.nih.gov/Taxonomy/Browser/wwwtax.cgi?id=4043) Pc4CL2  X13325.1 | MGDCVAPKEDLIFRSKLPDIYIPKHLPLHTYCFENISKVGDKSCLINGATGETFTYSQVELLSRKVASGLNKLGIQQGDTIMLLLPNSPEYFFAFLGASYRGAISTMANPFFTSAEVIKQLKASLAKLIITQACYVDKVKDYAAEKNIQIICIDDAPQDCLHFSKLMEADESEMPEVVIDSDDVVALPYSSGTTGLPKGVMLTHKGLVTSVAQQVDGDNPNLYMHSEDVMICILPLFHIYSLNAVLCCGLRAGVTILIMQKFDIVPFLELIQKYKVTIGPFVPPIVLAIAKSPVVDKYDLSSVRTVMSGAAPLGKELEDAVRAKFPNAKLGQGYGMTEAGPVLAMCLAFAKEPYEIKSGACGTVVRNAEMKIVDPETNASLPRNQRGEICIRGDQIMKGYLNDPESTRTTIDEEGWLHTGDIGFIDDDDELFIVDRLKEIIKYKGFQVAPAELEALLLTHPTISDAAVVPMIDEKAGEVPVAFVVRTNGFTTTEEEIKQFVSKQVVFYKRIFRVFFVDAIPKSPSGKILRKDLRAKIASGDLPK | | |
| *Arabidopsis thaliana* At4CL1  AY099747.1 | MAPQEQAVSQVMEKQSNNNNSDVIFRSKLPDIYIPNHLSLHDYIFQNISEFATKPCLINGPTGHVYTYSDVHVISRQIAANFHKLGVNQNDVVMLLLPNCPEFVLSFLAASFRGATATAANPFFTPAEIAKQAKASNTKLIITEARYVDKIKPLQNDDGVVIVCIDDNESVPIPEGCLRFTELTQSTTEASEVIDSVEISPDDVVALPYSSGTTGLPKGVMLTHKGLVTSVAQQVDGENPNLYFHSDDVILCVLPMFHIYALNSIMLCGLRVGAAILIMPKFEINLLLELIQRCKVTVAPMVPPIVLAIAKSSETEKYDLSSIRVVKSGAAPLGKELEDAVNAKFPNAKLGQGYGMTEAGPVLAMSLGFAKEPFPVKSGACGTVVRNAEMKIVDPDTGDSLSRNQPGEICIRGHQIMKGYLNNPAATAETIDKDGWLHTGDIGLIDDDDELFIVDRLKELIKYKGFQVAPAELEALLIGHPDITDVAVVAMKEEAAGEVPVAFVVKSKDSELSEDDVKQFVSKQVVFYKRINKVFFTESIPKAPSGKILRKDLRAKLANGL | | |
| *Arabidopsis thaliana* 4CL2  BT000296.1 | MTTQDVIVNDQNDQKQCSNDVIFRSRLPDIYIPNHLPLHDYIFENISEFAAKPCLINGPTGEVYTYADVHVTSRKLAAGLHNLGVKQHDVVMILLPNSPEVVLTFLAASFIGAITTSANPFFTPAEISKQAKASAAKLIVTQSRYVDKIKNLQNDGVLIVTTDSDAIPENCLRFSELTQSEEPRVDSIPEKISPEDVVALPFSSGTTGLPKGVMLTHKGLVTSVAQQVDGENPNLYFNRDDVILCVLPMFHIYALNSIMLCSLRVGATILIMPKFEITLLLEQIQRCKVTVAMVVPPIVLAIAKSPETEKYDLSSVRMVKSGAAPLGKELEDAISAKFPNAKLGQGYGMTEAGPVLAMSLGFAKEPFPVKSGACGTVVRNAEMKILDPDTGDSLPRNKPGEICIRGNQIMKGYLNDPLATASTIDKDGWLHTGDVGFIDDDDELFIVDRLKELIKYKGFQVAPAELESLLIGHPEINDVAVVAMKEEDAGEVPVAFVVRSKDSNISEDEIKQFVSKQVVFYKRINKVFFTDSIPKAPSGKILRKDLRARLANGLMN | | |
| *Arabidopsis thaliana* 4CL3  AY376730.1 | MITAALHEPQIHKPTDTSVVSDDVLPHSPPTPRIFRSKLPDIDIPNHLPLHTYCFEKLSSVSDKPCLIVGSTGKSYTYGETHLICRRVASGLYKLGIRKGDVIMILLQNSAEFVFSFMGASMIGAVSTTANPFYTSQELYKQLKSSGAKLIITHSQYVDKLKNLGENLTLITTDEPTPENCLPFSTLITDDETNPFQETVDIGGDDAAALPFSSGTTGLPKGVVLTHKSLITSVAQQVDGDNPNLYLKSNDVILCVLPLFHIYSLNSVLLNSLRSGATVLLMHKFEIGALLDLIQRHRVTIAALVPPLVIALAKNPTVNSYDLSSVRFVLSGAAPLGKELQDSLRRRLPQAILGQGYGMTEAGPVLSMSLGFAKEPIPTKSGSCGTVVRNAELKVVHLETRLSLGYNQPGEICIRGQQIMKEYLNDPEATSATIDEEGWLHTGDIGYVDEDDEIFIVDRLKEVIKFKGFQVPPAELESLLINHHSIADAAVVPQNDEVAGEVPVAFVVRSNGNDITEEDVKEYVAKQVVFYKRLHKVFFVASIPKSPSGKILRKDLKAKL | | |
| *Nicotiana tabacum* Nt4CL2  U50846.1 | MEKDTKQVDIIFRSKLPDIYIPNHLPLHSYCFENISEFSSRPCLINGANKQIYTYADVELNSRKVAAGLHKQGIQPKDTIMILLPNSPEFVFAFIGASYLGAISTMANPLFTPAEVVKQAKASSAKIIVTQACHVNKVKDYAFENDVKIICIDSAPEGCLHFSVLTQANEHDIPEVEIQPDDVVALPYSSGTTGLPKGVMLTHKGLVTSVAQQVDGENPNLYIHSEDVMLCVLPLFHIYSLNSVLLCGLRVGAAILIMQKFDIVSFLELIQRYKVTIGPFVPPIVLAIAKSPMVDDYDLSSVRTVMSGAAPLGKELEDTVRAKFPNAKLGQGYGMTEAGPVLAMCLAFAKEPFEIKSGACGTVVRNAEMKIVDPKTGNSLPRNQSGEICIRGDQIMKGYLNDPEATARTIDKEGWLYTGDIGYIDDDDELFIVDRLKELIKYKGFQVAPAELEALLLNHPNISDAAVVPMKDEQAGEVPVAFVVRSNGSTITEDEVKDFISKQVIFYKRIKRVFFVDAIPKSPSGKILRKDLRAKLAAGLPN | | |
| *Angelica decursiva*  Ad4CL | MGDCAAPKDIIFRSKLPDIYIPKHLPLHTYCFENISKVGDKACLINGATGETFTYSQVELLSRKVASGLNKLGIGQGDTIMLLLPNSPEYFFAFLGASYRGAISTMANPFFTSAEVIKQLKASQAKLIITQACYVDKVKDFATEQNIQIICIDDAPQGCLHFSKLMEADESEMPRVVINSDDVVALPYSSGTTGLPKGVMLTHKGLVTSVAQQVDGENPNLYIHSEDVMICILPLFHIYSLNAVLCCGLRAGATILIMQKFDIVPFLELIQKYKVTIGPFVPPIVLAIAKSPVVDKYDLSSVRTVMSGAAPLGKELEDAVRAKFPNAKLGQGYGMTEAGPVLAMCLAFAKEPYEIKSGACGTVVRNAEMKIVDPDTNASLPRNQRGEICIRGDQIMKGYLNDPESTKTTIDEEGWLHTGDIGFIDDDDELFIVDRLKEIIKYKGFQVAPAELEALLLTHPTISDAAVVPMIDEKAGEVPVAFVVRTNGFTTTEEEIKQFVSKQVVFYKRIFRVFFVDAIPKSPSGKILRKDLRARIASGDLPK | | |
| *Populus tomentosa* 4CL  AY043495.1 | MNPQEEFIFRSKLPDIYIPKNLPLHSYVLENLSNHSSKPCLINGANGDVYTYADVELTARRVASGLNKIGIQQGDVIMLFLPSSPEFVLAFLGASHRGAIITAANPFSTPAELAKHAKASRAKLLITQACYYEKVKDFARESDVKVMCVDSAPDGCLHFSELTQADENEAPQVDISPDDVVALPYSSGTTGLPKGVMLTHKGLITSVAQQVDGDNPNLYFHSEDVILCVLPMFHIYALNSIMLCGLRVGAPILIMPKFEIGSLLGLIEKYKVSIAPVVPPVMMSIAKSPDLDKHDLSSLRMIKSGGAPLGKELEDTVRAKFPQARLGQGYGMTEAGPVLAMCLAFAKEPFDIKPGACGTVVRNAEMKIVDPETGASLPRNQPGEICIRGDQIMKGYLNDPEATSRTIDKEGWLHTGDIGYIDDDDELFIVDRLKELIKYKGFQVAPAELEALLIAHPEISDAAVVGLKDEDAGEVPVAFVVKSEKSQATEDEIKQYISKQVIFYKRIKRVFFIEAIPKAPSGKILRKNLKEKLAGI | | |
| *Populus tremuloides* Pt4CL2  AF041050.1 | MMSVATVEPPKPELSPPQNQNAPSSHETDHIFRSKLPDITISNDLPLHAYCFENLSDFSDRPCLISGSTGKTYSFAETHLISRKVAAGLSNLGIKKGDVIMTLLQNCPEFVFSFIGASMIGAVITTANPFYTQSEIFKQFSASRAKLIITQSQYVNKLGDSDCHENNQKPGEDFIVITIDDPPENCLHFNVLVEASESEMPTVSILPDDPVALPFSSGTTGLPKGVILTHKSLITSVAQQVDGEIPNLYLKQDDVVLCVLPLFHIFSLNSVLLCSLRAGSAVLLMQKFEIGSLLELIQKHNVSVAAVVPPLVLALAKNPLEANFDLSSIRVVLSGAAPLGKELEDALRSRVPQAILGQGYGMTEAGPVLSMCLAFSKQPFPTKSGSCGTVVRNAELKVIDPETGRSLGYNQPGEICIRGSQIMKGYLNDAEATANTIDVEGWLHTGDIGYVDDDDEIFIVDRVKEIIKFKGFQVPPAELEALLVNHPSIADAAVVPQKDEVAGEVPVAFVVRSDDLDLSEEAVKEYIAKQVVFYKKLHKVFFVHSIPKSASGKILRKDLRAKLATATTMS | | |
| *Populus trichocarpa* Pt4CL3  EU603298.1 | MDAIMNSQEEFIFRSKLPDIYIPKNLPLHSYVLENLSKYSSKPCLINGANGDVCTYADVELTARRVASGLNKIGIQQGDVIMLFLPSSPEFVLAFLGASHRGAIVTAANPFSTPAELAKHAKASRAKLLITQACYYDKVKDFARESDVKVMCVDSAPDGCLHFSELTQADENEVPQVDFSPDDVVALPYSSGTTGLPKGVMLTHKGLITSVAQQVDGDNPNLYFHSEDVILCVLPMFHIYALNSIMLCGLRVGASILIMPKFDIGTLLGLIEKYKVSIAPVVPPVMLAIAKSPDFDKHDLSSLRMIKSGGAPLGKELEDTVRAKFPQARLGQGYGMTEAGPVLAMCLAFAKEPFDIKPGACGTVVRNAEMKIVDPETGASLPRNQPGEICIRGDQIMKGYLNDPEATSRTIDKEGWLHTGDIGYIDDDDELFIVDRLKELIKYKGFQVAPAELEALLLAHPEISDAAVVGMKDEDAGEVPVAFVVKSEKSQATEDEIKQYISKQVIFYKRIKRVFFIEAIPKAPSGKILRKNLRETLPGI | | |
| *Populus trichocarpa* Pt4CL5  EU603299.1 | MDTITKQKEEFIFRSKLPDIDIPKGLPLHSYVFENFSKYPSKPCLINGANGDVYTYADVELTARRAASGLNKLGIQQGDVIMLILPSSPEFVLAFLGASHRGAITTAANPFSTPAELAKQAKASKAKLLITQACYYDKVKDYAQQNDVKVMCVDSAPDVCLHFSELTQADDNDMPQVDIRPDDVVALPYSSGTTGLPKGVMLTHKGLITSVAQQVDGDNPNLYFHSEDVILCVLPMFHIYALNSIMLCGLRVGAAILIMPKFEIGSLLGLIEKYKVSIAPVVPPVMVAIAKSPDLDKHDLSSLRMLKSGGSPLGKELEDTVRARFPQARLGQGYGMTEAGPVLAMCLAFAKEPFDIKPGACGTVVRNAEMKIVDPETGSSLPRNLPGEICIRGDQIMKGYLNDPEATSRTIDNDGWLHTGDIGFIDDDDELFIVDRLKELIKYKGFQVAP  AELEALLQAHTGISDAAVVGMKDENSGEIPVAFVIKSENSQVTGEEIMQYISKQVIYYKKIKRVFFVEAIPKAPSGKILRKNLRERLAGGLQK | | |
| *Vitis vinifera* Vv4CL2  XM_002269909.2 | MEATQEFIYRSKLPDINIPSHLPLHSYCFQHISKFSSNPCLINAGNGNIYTYADVHLTARKVAAGLNRLGIRQGDAIMLLLQNCPEFVFSFLGASYLGATSTTANPFYTPAEIEKQATASSARIIITQACFAEKVKKFAEENDVKIICIDEPVAGCLHFSELSQADENDIPDVNISPDDVVALPYSSGTVGLPKGVMLTHKSMITSVAQQVDGENPNLYFHLDDVILCVLPMFHIYSLSSVVLCGLRVGAAILIMQKFEINTLMELVQKHKVTIAPFVPPILLAIAKSPVAHQYDLSSIRTVISGAAPMGKELEDSLGSKLPNAVIGQGYGMTEAGPVLSMCLAFAKEPFEIKSGACGTVVRNAEMKIINPETGASLPPNQAGEICIRGDQIMKGYLNDIEATKEAIDEEKWLHTGDIGYIDDNDELFIVDRLKEIIKNRGFQVAPAELEAILIAHPNIVDAAVVPMKDEAAGEVPIAFIVRSNGFEITEDEIKEYIAKQVVYYKRIKRVFFIETIPKAPSGKILRKDLKAKLAAGFSN | | |
| *Musa acuminata* Ma4CL3  XM_009397951.1 | MGSYSMPEETTFRSKLPDIDINNRRPLHAYCFERLADFADRPCIIDGASGAVMSYADVDIAARRAAAGLHRLGVGRGQVIMILLRNSPEFVLAFLAASHCGAVATTANPFYTPAEIHKQAAASGARVIVTESCYVDKVREFAQERGVTIVCADGPSEGCRHFSELLDADERDLHEVDIDPDDVVALPYSSGTTGLPKGVMLTHRSLITSVAQQVDGDNPNLYFHKEDVLLCVLPLFHIYSLNSVLLCGLRVGAAILIMRKFEISAMLELVQRYRVTVAPLVPPIVLDFVKSPLVDSYDLSSIRTVMSGAAPMGKELEDKFMAKLPNATLGQGYGMTEAGPVLSMCLAFAKEPFPVKSGACGTVVRNAELKIVDPDTGASLGHNKRGEICIRGAQIMKGYLNDPEATRNTIDKEGWLHTGDIGLVDDDDEIFIVDRLKELIKYKGFQVAPAELEALLITHHDIADAAVVPMKDELAGEVPVAFVVRCNGSQVTEEEIKQYVSKQVVFYKRINKVFFTEAIPKAPSGKILRKDLRAKLASPFPSA | | |
| *Physcomitrella patens*  Pa4CL4  EU180602.1 | MSPSVMSEPIMSESESRNKSIKPVQSVIRERATDRMVVKPMQSKVGDFIYRSKLPDIDIPNHMPLSDYCLEKAAQWPDKICLIDGVTGREYTYGEIELSTRRVAAGLFKIGVKQGDVIALLLPNCAEFVQVFLGAAKMGAIVTTANPFYTSAELEKQTIASGAGIVVTQSSYIEKLAGLNLQIITVDHHVEKCMHISMLLEADEAECPQVEIHPDDVVCLPYSSGTTGLPKGVMLTHKSLVSSVSQQVDGDSPNFSITVEDTLMCVLPMFHIYSLNSILLCGLRVGATLVIMPKFELSKMLELIQNHKVTMGPFVPPIVLAIAKNPMVENYDLSSIKMVMSGAAPLGKELEDAFRGRLPNAILGQGYGMTEAGPVLAMCLAFAKSPFPVKPGSCGTVVRNAEVKIVDTETGMSLPYNQPGEICIRGPQIMKGYLNNPEATANTIDKDGFLHTGDVAFIDEDEEMFIVDRVKEIIKFKGFQVPPAELEALLLSNEEIQDAAVVSRKDDVAGEVPVAFVVRQAGSTISEEEVKDYVAKQVVFYKEIRNVYFVDSIPKSPSGKILRKDLRNKV | | |
| *Oryza sativa* 4CL  X52623.1 | MGSMEQQQPESAAPATEASPEIIFRSKLQDIAITNTLPLHRYCFERLPEVAARPCLIDGATGGVLTYADVDRLSRRLAAALRRAPLGLRRGGVVMSLLRNSPEFVLSFFAASRVGAAVTTANPMSTPHEIESQLAAAGATVVITESMAADKLPSHSHGALTVVLIDERRDGCLHFWDDLMSEDEASPLAGDEDDEKVFDPDDVVALPYSSGTTGLPKGVMLTHRSLSTSVAQQVDGENPNIGLHAGDVILCALPMFHIYSLNTIMMCGLRVGAAIVVMRRFDLAAMMDLVERHRVTIAPLVPPIVVAVAKSEAAAARDLSSVRMVLSGAAPMGKDIEDAFMAKLPGAVLGQGYGMTEAGPVLSMCLAFAKEPFKVKSGACGTVVRNAELKIIDPDTGKSLGRNLRGEICIRGQQIMKGYLNNPEATKNTIDAEGWLHTGDIGYVDDDDEIFIVDRLKEIIKYRGFQVAPAELEALLNTHPSIADAAVVGLKFGEIPVAFVAKTEGSELSEDDVKQFVAKEVIYYKKIREVFFVDKIPKAPSGKILRKELRKQLQHLQQEALTN | | |
| *Glycine max* Gm4CL1  NP_001236418.1 | MADDGSRRELIFRSKLPDIYIPKHMPLHSYCFENLRECGSRPCLINAPTGDVYSYHEVDSTARKVARGLKKEGVEQGQVIMILLPNCPEFVFSFLGASHRGAMATAANPFFTPAEIAKQAHASNAKLLITQASYYDKVKDLRDIKLVFVDSCPPHTEEKQHLHFSHLCEDNGDADVDVDVDIKPDDVVALPYSSGTTGLPKGVMLSHKGLVTSIAQQVDGDNPNLYYHCHDTILCVLPLFHIYSLNSVLLCGLRAKATILLMPKFDINSLLALIHKHKVTIAPVVPPIVLAISKSPDLHKYDLSSIRVLKSGGAPLGKELEDTLRAKFPNAKLGQGYGMTEAGPVLTMSLAFAKEPIDVKPGACGTVVRNAEMKIVDPETGHSLPRNQSGEICIRGDQIMKGYLNDGEATERTIDKDGWLHTGDIGYIDDDDELFIVDRLKELIKYKGFQVAPAELEALLLTHPKISDAAVVPMKDEAAGEVPVAFVVISNGYTDTTEDEIKQFISKQVVFYKRINRVFFIDAIPKSPSGKILRKDLRAKIAASVPK | | |
| *Glycine max* Gm4CL4  CAC36095.1 | MITLAPSLDTPKTDQNQVSDPQTSHVFKSKLPDIPISNHLPLHSYCFQNLSQFAHRPCLIVGPASKTFTYADTHLISSKIAAGLSNLGILKGDVVMILLQNSADFVFSFLAISMIGAVATTANPFYTAPEIFKQFTVSKAKLIITQAMYVDKLRNHDGAKLGEDFKVVTVDDPPENCLHFSVLSEANESDVPEVEIHPDDAVAMPFSSGTTGLPKGVILTHKSLTTSVAQQVDGENPNLYLTTEDVLLCVLPLFHIFSLNSVLLCALRAGSAVLLMQKFEIGTLLELIQRHRVSVAMVVPPLVLALAKNPMVADFDLSSIRLVLSGAAPLGKELEEALRNRMPQAVLGQGYGMTEAGPVLSMCLGFAKQPFQTKSGSCGTVVRNAELKVVDPETGRSLGYNQPGEICIRGQQIMKGYLNDEAATASTIDSEGWLHTGDVGYVDDDDEIFIVDRVKELIKYKGFQVPPAELEGLLVSHPSIADAAVVPQKDVAAGEVPVAFVVRSNGFDLTEEAVKEFIAKQVVFYKRLHKVYFVHAIPKSPSGKILRKDLRAKLETAATQTP | | |
| *Zea mays* Zm4CL  AAS67644.1 | | MGSVDAAIAVPVPAAEEKAVEEKAMVFRSKLPDIEIDSSMALHTYCFGKMGEVAERACLIDGLTGASYTYAEVESLSRRAASGLRAMGVGKGDVVMSLLRNCPEFAFTFLGAARLGAATTTANPFYTPHEVHRQAEAAGARLIVTEACAVEKVREFAAERGIPVVTVDGRFDGCVEFAELIAAEELEADADIHPDDVVALPYSSGTTGLPKGVMLTHRSLITSVAQQVDGENPNLYFRKDDVVLCLLPLFHIYSLNSVLLAGLRAGSTIVIMRKFDLGALVDLVRRYVITIAPFVPPIVVEIAKSPRVTAGDLASIRMVMSGAAPMGKELQDAFMAKIPNAVLGQGYGMTEAGPVLAMCLAFAKEPYPVKSGSCGTVVRNAELKIVDPDTGAALGRNQPGEICIRGEQIMKGYLNDPESTKNTIDQDGWLHTGDIGYVDDDDEIFIVDRLKEIIKYKGFQVPPAELEALLITHPEIKDAAVVSMNDDLAGEIPVAFIVRTEGSQVTEDEIKQFVAKEVVFYKKIHKVFFTESIPKNPSGKILRKDLRARLAAGVH |  |
|  |  | | |
